# What is esports? A systematic scoping review and concept analysis of esports

**DOI:** 10.1016/j.heliyon.2023.e23248

**Published:** 2023-12-03

**Authors:** Di Tang, Raymond Kim-wai Sum, Minghui Li, Ruisi Ma, Peichi Chung, Ray Wai-keung Ho

**Affiliations:** a Department of Sports Science and Physical Education, Faculty of Education, The Chinese University of Hong Kong, Hong Kong, China; b Department of Cultural and Religious Studies, Faculty of Arts, The Chinese University of Hong Kong, Hong Kong, China; c Centre for Physical Activity, Sport and Exercise Sciences, Coventry University, Coventry, United Kingdom

**Keywords:** Esports, Scoping review, Concept analysis, Definition, Thematic analysis

## Abstract

This study conducted a systematic scoping review to collect definitions and explanations of esports and its related concepts in the existing literature. A thematic coding approach was used to screen possible disagreements and issues between the different characteristics of esports as presented in the existing definitions. Based on a critical discussion, the results of the coding were combined with the objective facts of the current esports industry and theories from similar fields to reconstruct the construction of an esports definition. In addition, the relationship between esports and its related concepts was discussed based on the proposed esports definition structure. The proposed esports criteria can help avoid misunderstandings and confusion in future studies regarding the research subjects related to esports and propose standardized usage of relevant terms in the field of esports research.

## 1 Introduction

Since 2010, the esports industry has seen tremendous growth, driven by the increase in game players and audiences and technology development [1,2]. With the emergence of its economic value, esports has started to receive great attention from various fields. However, the development of esports theory and scientific research has been lagging behind the development of practice. In terms of the number of studies, from 2002 to March 2018, less than 200 articles on esports were published in various research fields from four databases [3], and it was not until 2020 that the quantity of esports research started to increase significantly [4,5]. However, there has been no widely accepted definition for esports [6]. This situation also causes a series of problems in esports research and development: (1) The varying definitions of esports exhibit conspicuous differences and even inconsistencies, engendering a degree of misunderstanding and perplexity in comprehending the results of diverse esports investigations. The generalization of findings across diverse esports investigations is hindered by the absence of consensus regarding the identification of esports. For example, in a recent study, we can see that the result of research on massively multiplayer online role-playing games (MMORPG) was assimilated into the domain of esports [7], although it is not conventionally regarded as esports. (2) In some esports-related research articles, because different definitions are somewhat controversial, these articles only list the differences and development of multiple esports concepts instead of giving a certain adopted definition [[8], [9], [10]]. Therefore, without a precise definition of the research subjects, there will be certain obstacles to the generalization and application of the conclusions of these studies. (3) The existence of multiple alternative and related concepts without a uniform definition and use standard [[11], [12], [13], [14], [15]] will result in the misuse of these concepts, and misunderstandings of the conclusions of the related research. Therefore, clarifying the concept of esports and the relationship between esports and its related concepts is an important basis for future research, and it is also a necessary part of improving public awareness of esports and promoting the development of the esports industry. Additionally, esports has become a formal competition in Asian Games in 2022, and what games were selected by Asian Games will also provide new insights from the industry on how we define esports. Since 2016, an increasing number of new esports games, such as mobile games (e.g., Honor of King) and motion sensing games (e.g., Just Dance, Formula One Esports Series), have appeared in the formal international esports competition. All of this brings us to the necessity to revisit and understand the definitions of esports in the context of industry trends.

Despite the ongoing controversies, there has yet to be a systematic evaluation of these divergent perspectives. To address this issue, the present study employs a scoping review approach to summarize, evaluate, and discuss the relevant definitions and research on esports. Scoping reviews employ clear, precise, and transparent research methods to minimize bias and provide a comprehensive overview of current knowledge [16,17], and a systematic review aims to “map the key concepts underpinning a research area and the main sources and types of evidence available [18]”, which is applicable to addressing the current issues in the field of esports research. This study aimed to systematically collect, identify, and analyze the core attributes of esports and screen potential areas of contention within the definitions present across various literature sources. We wanted to explore and understand the commonalities and contradictions between the different definitions and see if the various definitions of esports could explain the esports we see in the industry to this day. By systematically summarizing the commonalities and contradictions among these different definitions, preserving the features and content that have reached consensus, and understanding and resolving the points of contradiction, we can integrate a framework for the concept of esports that is in line with the current situation. This framework can provide clearer standards for establishing the research object of future studies related to esports. In addition, integrating the current academic explanations and discussions of similar concepts in esports will construct a more systematic and organized terminology system related to esports. Through such efforts, it is plausible that we can effectively address the aforementioned limitations and issues inherent in contemporary esports research.

## 2 Methods

### 2.1 Research objective

In this study, we conducted a scoping review to:

(1) Collect, analyze, and evaluate the core attributes of esports from the existing literature, identify the potential issues under each theme through thematic coding, and finally propose a comprehensive framework of esports in line with the background of the contemporary era;
(2) Clarify several related concepts in the field of esports research based on views and trends in the literature and delineate how these concepts relate to esports.

### 2.2 Protocol and registration

The protocol of this scoping review was registered on the Open Science Framework (OSF: https://doi.org/10.17605/OSF.IO/NYX6D).

### 2.3 Search strategy

An electronic search strategy was employed using the following databases: (1) SPORTDiscus, (2) Scopus, (3) Web of Science, and (4) PubMed. No particular start date was adopted, and the last search was conducted on 13 May 2022. These databases are related to sports, education, sociology, information technology, and health, which are suitable for the topic of esports and increase the probability that all relevant studies (in English) have been located. The boolean logic combinations search strategy was adopted within the electronic databases, including “electronic sport(s)”, esport(s), “e-sport(s)”, cybersport(s), “cyber sport(s)”, “virtual sport(s)”, and “digital sport(s)”.

### 2.4 Eligibility criteria

The inclusion criteria of this scoping review were:

(1) Articles containing the definition or explanation of the concept of esports;
(2) Articles with published status;
(3) Publications in English;

The exclusion criteria of this scoping review were:

(1) Keywords in search strategies such as esports appear in the title, keywords, or abstract of the article, but there is no clear explanation of the definition of esports given in the full text;
(2) Not in full-text format.

Since some of the mainstream and important definitions of esports come from different types of publications, including conference papers, book chapters, statements from international organizations, etc., and there is no unified document type filter across the different databases, in the initial document search, the document type was not limited. After reading the literature at a later stage, the publication types of the selected literature were summarized.

This study followed the guidelines of Preferred Reporting Items for Systematic Reviews and Meta-Analysis extension for scoping reviews (PRISMA-ScR) [19]. The details of the PRISMA-ScR checklist are provided in Appendix A. Two main reviewers worked independently on screening and data exaction. The criteria were discussed by two reviewers for accuracy before the formal process, and a pilot test was done with ten articles to refine the standards and reach a consensus on the practice of screening and data extraction. Any disagreements were resolved through discussion between the two reviewers or further adjudication by a third reviewer. In accordance with the procedures, a total of 1590 duplicate papers were removed. After removing all the duplicates, the papers were read thoroughly and screened based on the inclusion and exclusion criteria. Additional records were also added to the identified process (obtained from the references of the reviewed articles that did not appear in the search process in the selected databases). A total of 54 articles were added to the additional records for the identified process. In the end, 299 articles were included.

### 2.5 Data exaction and qualitative synthesis

A close re-reading of 299 literature sources was done in the initial stage of analysis as a familiarization process. We extracted the definitions of esports adopted by the papers and any other material describing the concept's different dimensions from the included literature. Thereafter, thematic coding was used to identify categories from the papers in the analysis. Thematic coding is a method of identifying common themes within passages of text with the purpose of data retrieval [20]. A three-step coding was performed in the process, starting from an open coding. The intention behind the whole process was to extract the core attribute from the initial coding. To assist with this process, NVivo 12 was used to organize the data. Two independent coders participated in the process of thematic coding. The two coders exhibited a noteworthy level of agreement, achieving 91 % intercoder agreement, which surpasses the recommended threshold of 85 % as proposed [21]. Cohen's kappa (κ) was also calculated to increase the credibility of the research. Cohen's kappa (κ) assesses the overall reliability between coders, with values closer to 1 indicating stronger agreement [22]. An excellent level of agreement is indicated by a Cohen's kappa (κ) value exceeding 0.8. In this study, the calculated Cohen's kappa (κ) value is 0.90.

#### 2.5.1 Procedure of qualitative analysis

**Fig. 1:**
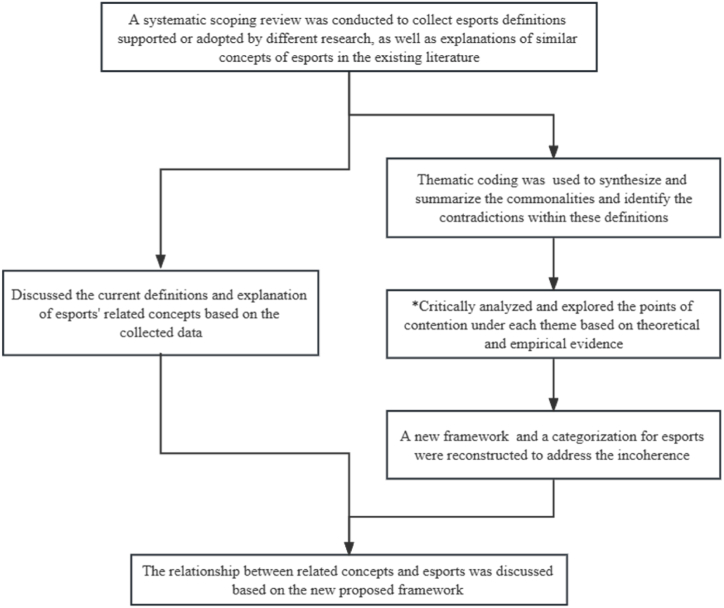
The process of qualitative analysis.

A flow chart (Fig. 1) illustrates the entire qualitative analysis process. Through the process of thematic coding, we identify and extract the underlying themes or characteristics of esports reflected in these diverse definitions and explanations. Subsequently, we synthesize and summarize the commonalities within these different themes, as well as identify the contradictions and points of controversy that emerge within specific themes. In the critical analysis of contradictions, when different points of contention or contradiction were encountered under the same theme, the discussion was based on the following criteria:

(1) Analysis of the text of the definitions to identify if there is any linguistic ambiguity in the definition or interpretation of esports or controversies caused by linguistic differences between countries (e.g., spelling, differences in literal explanation, etc.）
(2) The number and proportion of each theme or feature supported or mentioned by different studies were used as evidence for a discussion on whether a particular feature has reached a dominance in interpretation or whether the idea is already well supported by the structured theory.
(3) Whether this feature explains the shape and character of esports as recognized in the industry today, such as the esports events involved in the Asian Games and emerging esports activities recognized by the current esports community or media (Just Dance, Formula One Esports Series). The ability of this feature to explain the esports currently being proposed in the community was also an important criterion [23].

#### 2.5.2 Concept analysis

In addition to thematic coding, in this study, we also referenced part of the framework of concept analysis to discuss and analyze the concept of esports. Concept analysis is regarded as a method to explore the attributes of a concept and clarify its definition. This study referenced Rodgers’ six-step method concept analysis (see Table 1) [24], which was selected for the following reasons: (1) It is an inductive approach that supported the fact that concepts are dynamic in nature, as they continuously develop across both time and varying contexts; (2) The philosophical basis for this method rejects the existence of a “crystal clear” notion of what a concept is [24]; (3) It meets the research aims of this study (to clarify alternative and related concepts of esports and to summarize and analyze the attributes of esports).

**Table 1:** Rodgers’ evolutionary concept analysis [24].

(1) Identify the name and concept of interest and association expressions (including surrogate terms).
(2) Identify and select the appropriate realm (setting and sample) for data collection.
(3) Collect relevant data to identify the attributes of the concept and the contextual basis of the concept, including interdisciplinary, sociocultural, and temporal (antecedents and consequential occurrences) variations.
(4) Analyze data regarding the above characteristics of the concept.
(5) Identify an exemplar of the concept, if appropriate.
(6) Identify implications, hypotheses, and implications for further development of the concept.

## 3 Results

### 3.1 Summary

**Fig. 2:**
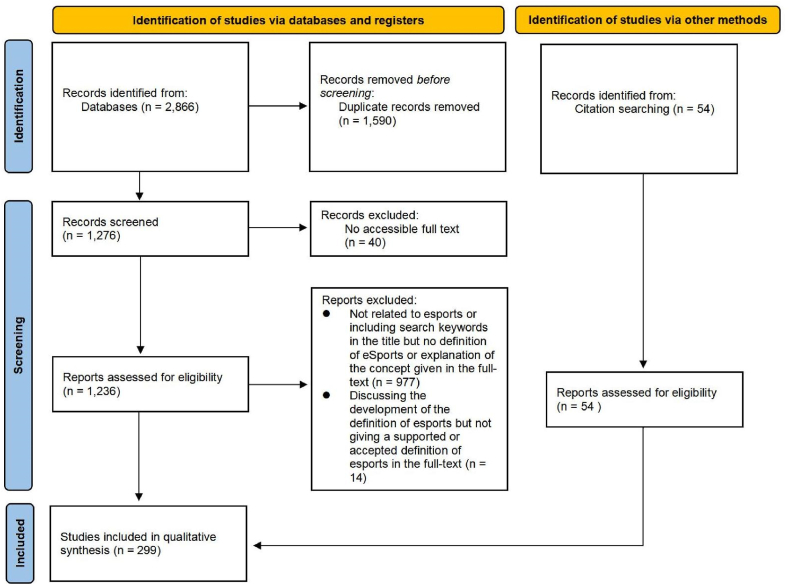
A flow diagram of identification and selection.

The number of papers that were identified, screened, and considered for eligibility is summarized in Fig. 2. In the end, a total of 299 articles were identified as suitable for further qualitative synthesis, and these articles were divided into three types of publication: (1) book chapter, (2) journal article, and (3) conference papers.

**Fig. 3:**
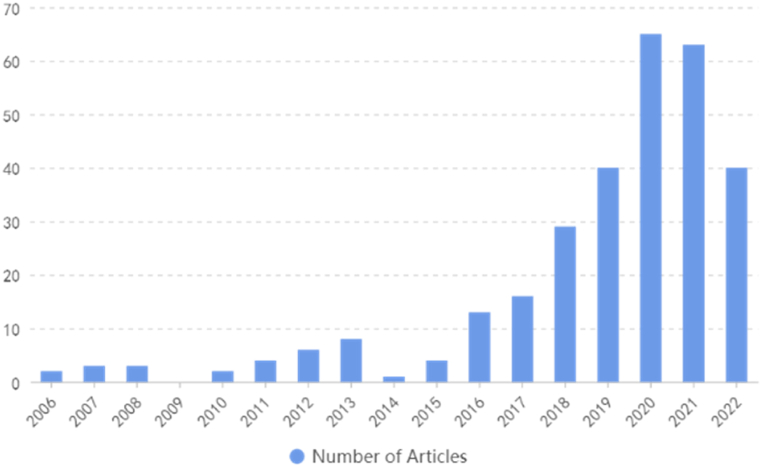
Year of publication.

The year of publication for these papers is displayed in Fig. 3. It is evident that starting from 2018, there has been an increasing amount of interpretation and discussion regarding the definition of esports. Furthermore, among the selected 299 papers, 47 of them cited the definition provided by Hamari [2], while 30 papers cited the definition by Wagner [25,26]. Table 2 illustrates the most frequently cited definitions within the 299 papers.

**Table 2:** Most-cited esports definitions.

Article	Definition	Citations
Wagner (2007)	An area of sport activities in which people develop and train mental or physical abilities in the use of information and communication technologies [26].	5
Freeman (2017)	Esports as computer-mediated “sports”, as competitive computer gaming, as spectatorship (three core attributes were given in the article) [27].	7
Witkowski (2012)	Esports commonly refer to an organized and competitive approach to playing computer games [28].	7
Wagner (2006)	Esports is an area of sport activities in which people develop and train mental or physical abilities in the use of information and communication technologies [25].	25
Pedraza-Ramirez (2020)	Esports is the casual or organized competitive activity of playing specific video games that provide professional and/or personal development to the player. This practice is facilitated by electronic systems, either computers, consoles, tablets, or mobile phones, on which teams and individual players practice and compete online and/or in local-area-network tournaments at the professional or amateur level. The games are established by ranking systems and competitions and are regulated by official leagues. This structure provides players with a sense of being part of a community and facilitates mastering expertise in fine-motor coordination and perceptual-cognitive skills, particularly but not exclusively, at higher levels of performance [29].	5
Hutchins (2008)	Organized competitive gaming [30].	5
Jenny (2017)	Organized video game competitions [31].	10
Hamari (2017)	A form of sports where the primary aspects of the sport are facilitated by electronic systems; the input of players and teams as well as the output of the esports system are mediated by human-computer interfaces [2].	47

### 3.2 Qualitative synthesis

The analysis identified a total of 1157 codes, which were organized into 96 core categories and then further organized into 19 subthemes. Finally, these subthemes were grouped into two higher-order themes. Table 3 presents the findings, with numbers in parentheses indicating the frequency of core categories used in the definitions across the 299 articles. It was observed that among these categories, besides certain attribution relationships, there were also contradictory relationships, which reflect the ongoing controversy and contradictions in defining esports. The subsequent section provides a comprehensive review of these core categories, subthemes, and higher-order themes. Each theme is then explained and analyzed, following the framework and steps of concept analysis.

**Table 3:** The hierarchical structure of coding results, including core categories, subthemes, and higher-order themes.

Core categories	Subthemes	Higher-order themes
Computer/video games (231)	Game	Intrinsic Properties
Competitive (159)	Competitiveness	
Competition (81)		
Overcome their opponents (3)		
Vie for superiority (1)		
Who can be the best in a specific game (1)		
Players competing against one another (3)		
Software and hardware devices (19)	Technology	
Virtual environment built using information technology (29)		
Electronically extended human actions (15)		
Computer-mediated or -generated (8)		
Science (1)		
Practiced entirely in digital form or mixed competitive entertainment (1)		
Equipment or apparatus manufactured for the main purpose of using the video material (1)		
Interactive tools (1)		
Multiplayer (31)	Participation	
Matching (1)		
Between people (1)		
Team and individually (28)		
Dual player (7)		
Players form teams (6)		
Widely attended (1)		
Remotely (2)		
Online (27)		
Head-to-head (6)		
Take place in person and/or in an interactive online environment (1)		
Assemble in stadia and arenas (1)		
A local area network (1)		
Played in real time (4)		
Asynchronous (1)		
Professional (52)	Level	
Training (1)		
Experts (3)		
Top level (2)		
Casual (2)		
Leisure (2)		
Entertainment (9)		
Serious (2)		
Semi-professionals (1)		
Sport (55)	Sportification	
Exercise (2)		
Movement (2)		
Train and develop mental and physical skills (19)	Skills or Literacy	
Hand-eye coordination skills (1)		
Optimizing esports performance (1)		
The embodied performances of players themselves (1)		
Skill-intensive (1)		
Intellectual (5)		
Not win by chance, but by utilizing their experience and skill sets (3)		
The contestants hardly move (1)		
Tactic control (1)		
Cooperative (2)		
Team spirit (1)		
A sense of being part of a community (1)		
Achieve a specific goal by the end of the game (8)	Goal	
Playing for trophies or points (1)		
Playing for speed (1)		
Improving the quality of players (1)		
A goal-oriented application (1)		
Individual achievement (1)		
Making a living (2)		
Prizes (9)		
Financially incentivized (1)		
35 different video games (1)	Game Genre	
Specific video games (1)		
Game genres (1)		
Reproducible game framework (1)		
Fighting games (2)		
Card games (2)		
Board games (1)		
Shooting games (2)		
A fair rating and evaluation (2)	Rating System	
Ranking systems (2)		
Emerging (6)	Emerging	
Modern (1)		
Obey the uniform rules (10)	Rules	
Fair (1)		
Carry out fair confrontation (1)		
Organized (66)	Organization	External Environment or Relationship
Coordinated by different leagues and tournaments (47)		
Ranging from top tier professional teams to local esports clubs and unions (2)		
Social organization (2)		
A governing body (3)		
Spectator (36)	Audience	
Streaming (4)		
In public (1)		
Sponsored by various business organizations (7)	Commercial	
Through promotional activities (1)		
Superstars, playoffs, fans, uniforms, comebacks, and upsets (1)		
Media (4)	Media	
Broadcasters (4)		
Culture (5)	Culture	
Cultural phenomenon (1)		
Industry (2)	Industry	
Infrastructure built around them (1)		
Whether or not a community frames it as an esports (1)	Acceptance	
Selected for official inclusion in an international esports competition (1)		

*Numbers in parentheses mean the frequency of the core categories used in the definitions in 299 articles.

#### 3.2.1 Surrogate terms and related concepts

Based on Rodger's theory of concept analysis, surrogate terms refer to alternative terms of the chosen concept, and related concepts are concepts in relation to the selected concept but are not the same concept [24]. However, due to the lack of clarity in the definition of esports, in this study, we analyzed surrogate terms and related concepts together, clarified these concepts, and discussed their differences and similarities based on the literature. The following terms were identified as target terms: electronic sports, e-sports, eSports, cybersports, cyber sports, digital sports, and virtual sports.

##### 3.2.1.1 Electronic sports, e-sports, eSports, and esports

First of all, it is generally accepted that esports is the abbreviation of electronic sports, and “eSports”, “esports”, and “e-sports” are only different in English writing. The term “esports” is often used in the Anglo-American area, and the Associated Press in America announced that “esports” is confirmed as the official spelling in the American Copy Editors Society meeting in the US in 2017 [29,32]; “eSports” is the prevailing spelling in Europe; and “e-Sports” is adopted in Asia [33]. In this article, they were considered the same term, and “esports” was adopted in this paper.

##### 3.2.1.2 Virtual sports

In the screened articles, 33 papers mentioned virtual sports. In 29 of these articles, virtual sports tends to be a sport or physical activity (such as dance) played in a virtual environment and utilizing information technology, especially motion capture, augmented reality (AR), and virtual reality (VR). Virtual sports in these papers highlights the body movement involved in the process. Besides, some articles even mentioned that in this kind of activity, electronically modified sports equipment might be used, such as “a real ball” or “a bat” [34,35]. Such a concept shares some similarities with exergaming, exergames, and active video games, which are emerging in recent sports or education research areas. Exergame commonly refers to video games in which physical effort is needed, and exergaming is “an experiential activity … which involves playing exergames or any videogames that require physical exertion or movements that are more than sedentary activities and also include strength, balance, and flexibility activities” [36]. Active video games refer to video games that require physical activity beyond that of conventional hand-controlled games [37].

By contrast, it is easy to see that exergames, active video games, and virtual sports all emphasize the physical element of the activities. Virtual sports emphasize the more traditional sports elements, while exergames show a wider of game types rather than being limited to imitating traditional sports. Virtual sports sometimes refers to sports video games [38,39]. In the searched articles, virtual sports were often used and discussed in the field of traditional sports training in the early days. With the advancement of technology, it has become possible to create an indoor virtual sports environment for sports training that accurately simulates real sports settings [40,41]. It is evident that exergames utilize advanced electronic equipment to enhance the variety of input methods and control mechanisms for players during the gameplay. Instead of relying solely on traditional input devices such as mice, keyboards, or buttons, motion capture, AR, and VR technology allows players to interact using various physical actions. Meanwhile, three articles directly equate virtual sports to esports. Considering the proportion of these viewpoints in the analyzed literature and the subsequent discussion on the definition of esports., we do not fully equate virtual sports to esports in this article.

Besides, three articles propose the concept of physical esports, specifying that physical esports “enables players to feel the actions involved in the actual sport not only through computer graphics (CG) images but also through feedback to their bodies using extended reality (XR) technology without being concerned about physical limitations or age-restricted.” [[42], [43], [44]]. Based on this concept, we found that virtual sports share some commonality with the definition of physical esports. Additionally, we can find some activities that fit the definition of virtual sports or physical esports start to appear in formal international esports competitions. For example, Formula One Esports Series has become one of the important esports tournaments since 2017; Just Dance became a formal competition in Electronic Sports World Cup (ESWC) in 2017 and 2019. The classification of virtual sports as esports, as well as the specific types of virtual sports that can be considered esports, still require further exploration and substantiation through additional theoretical and empirical evidence. Nonetheless, the current findings indicate the potential for such categorization.

##### 3.2.1.3 Cybersports and cyber sports

The terms cyber sports and cybersports in the analyzed literature are also only different in English writing. There is no difference between them. Fourteen articles mentioned the concept of cyber sports, of which 11 articles equate cyber sports to esports and three articles equate cyber sports to virtual sports. In this article, we took cyber sports as a surrogate term for esports.

##### 3.2.1.4 Digital sports

Among the reviewed articles that utilize the term “digital sports”, none of them provide a specific definition or explanation for the term. Therefore, in this section, we have compiled various perspectives and examples related to digital sports from the reviewed literature in order to summarize their interpretation of digital sports.

A total of 23 articles mentioned and discussed the term “digital sports” while offering various perspectives and examples. However, these articles differ from the ones mentioned earlier regarding terms such as virtual sports and cyber sports. Their interpretation of digital sports is entirely distinct, and there is no clear or prevailing consensus on its definition.

These views can be divided into four groups. Four of the 23 articles refer to digital sports as a concept similar to exergames or active video games, and another four articles refer to digital sports as sports computer games, such as EA sports games, racing games, etc. Five articles use digital sports to refer to physical exercise with modern technology equipment, such as fitness mobile applications, digital coaches, online sports platforms, etc. In these articles, the core component of digital sports is physical exercise in general, and it is not necessarily video games or competitive. Besides, among these 23 articles, 10 articles take digital sports as the digitalization of sports, and sometimes it means the digitalization of the whole sports industry:

(1) In a narrow sense, new information technology or electronic equipment is implemented in the training of traditional sports, such as utilizing electronic sensors in traditional sports training and some wearable devices to detect the real-time physiological state of athletes. It is called the transition from sports to digital sports.
(2) In a broader sense, digitalization encompasses the entire sports industry, extending beyond sports activities alone. It includes the application of information technology in modern electronic sports media, as well as the advancements in camera equipment and broadcasting technology in professional sports competitions. These developments collectively contribute to the transformation of sports into digital sports.

Hence, digital sports should be distinguished from the aforementioned esports-related terms as it does not represent a specific form of sports. This term will not be extensively differentiated from other terms. In this article, digital sports is considered to be traditional sports that have undergone a significant level of digitalization.

#### 3.2.2 Attributes

This step aims to extract a list of defining characteristics that gives broad insight into the concept and differentiates it from other phenomena [24]. This part is based on the thematic coding from the included literature.

##### 3.2.2.1 Intrinsic properties

###### 3.2.2.1.1 Game, rules, and goals

Of the 299 reviewed articles, 231 indicate that esports is computer games or video games. Therefore, it is widely acknowledged that esports originates from computer games and video games [31,45,46]. Among these 231 articles, 15 of them specifically define esports as “computer games”. Firstly, the term “computer games” can cause some ambiguity. In different contexts, computer games can refer to PC games installed on operating systems such as Windows or Mac OS, controlled and played using devices like keyboards, mice, or game controllers. It can also encompass video games with a broader range of platforms and control methods. Secondly, neither the related papers nor the current esports competitions provide any supporting evidence or theoretical reasoning as to why esports should be limited to PC games. It is evident that esports competitions include more than just PC games. Other forms of games (mobile games, motion-controlled games) have already been accepted by official international esports competitions, such as the World Cyber Games (WCG), Cyberathlete Professional League (CPL), and Asian Games. Therefore, the terms “video games” or “electronic games” might be more suitable for defining esports. Additionally, we have also witnessed the impact of technological changes and advancements on the definition of esports.

Besides, a total of 12 articles mention “rules” in their definition of esports. They highlight that esports should obey uniform rules and carry out fair confrontations. In fact, Juul lists six features of games, and he indicates that all the games should adopt one particular rule, which is an inherent attribute of games [47]. Furthermore, it was also proposed in the previous research that games are inherently goal-oriented activities [[48], [49], [50]]. Of the reviewed articles, there are also 26 that mentioned “goal” in the definition of esports. Their explanation of goals can be divided into two groups: goals within games and goals in real life. Four definitions demonstrate that esports is played for speed, points, or victory in the game, while in other definitions, the goal of esports is to “win prizes or make a living” in real life. Based on the definition of games, all the games have a goal to be achieved when played, which means the in-game goal. Therefore, “rules” and “goals” are the essential attributes of the game. In other words, recognizing esports as one video or electronic game implies accepting the feature of “rules” and “goals (within games)”. Hence, some of these codes overlap in meaning. In the existing literature, the purposes of esports in real life are primarily categorized into two types: one is using esports as a means of livelihood, and the other is self-entertainment. Furthermore, we have also come across new articles that utilize esports for educational purposes [[51], [52], [53]]. In traditional sports, the purposes of different sporting activities often serve as criteria for categorizing sports.

###### 3.2.2.1.2 Competitiveness, game genre, and rating system

A total of 248 articles underline “competitiveness” in their definitions of esports, including phrases such as “overcome their opponents”, “vie for superiority”, “competing against one another”, and so on. Competitiveness is an essential indicator to distinguish esports from general video games. Additionally, 11 articles also point out that game genre can be considered as an indicator to distinguish between esports and computer games. They list some game genres when defining esports, such as shooting games, fighting games, card games, and so on. In general, first-person shooter (FPS), multiplayer online battle arena (MOBA), real time strategy game (RTS), sports simulation games, card games, and fighting games are the mainstream and widely recognized genres in the current esports industry. These genres typically possess well-developed tournament structures and organized systems [[54], [55], [56], [57]]. Moreover, they have garnered recognition and inclusion in prominent international esports events. Additionally, these genres have been featured as distinct event categories within the realm of esports, even serving as medal events during the 2023 Asian Games [58]. Scholars have also proposed different categories for esports: imagination (e.g., DotA 2, League of Legends, Overwatch), physical enhancement (e.g., Counter-Strike: Global Offensive, Halo series), and sports simulation (e.g., FIFA series, NBA 2K series) [59]. It is obvious that all of these games are competitive, and conventionally, these games are considered as esports by the public and esports organizations, and they are conventional events in esports competitions. Besides, the rating system in the game also enhances the competitive nature of the game. In sum, distinguishing esports by game genre and rating system is essentially all about distinguishing esports from general video games through its competitive nature. Typically, this rating system in games manifests as a well-defined criterion or scoring mechanism that distinguishes wins and losses, similar to traditional sports. However, with the continuous iteration and progress of games, this rating system has also evolved into an evaluation system for player performance and skill level [60,61]. For instance, in FPS and MOBA games, it may include metrics such as kill count, ranking system in MOBA ranking games, and shooting accuracy in FPS. These refined quantitative indicators further enhance the competitiveness among players. According to the proportion of the different codes, “game” and “competitiveness” are the most essential properties in the definition of esports. Therefore, we also found that the majority of articles take competitive computer games or video games as the common definition of esports.

###### 3.2.2.1.3 Technology

Most of the articles acknowledge that “e” in esports represents “electronic” [2,33,62]. Fifty-six out of 299 articles discussed “technology” in the definition of esports, which is related to the term “electronic” in esports. “Technology” in the reviewed articles usually means the use of information and communication technologies in games, which realizes the interaction between people and computers or devices and interactions between players. Meanwhile, the continuous advancements in these technologies are constantly enriching the input forms for players (beyond just keyboards and mice) and the output forms of computers or other devices during gameplay (beyond just computer screens). For example, the maturity of mobile technology and mobile game design have led to the gradual inclusion of mobile games in formal esports competitions. For example, in the upcoming Asian Games, two mobile games have been selected for esports medal competitions; Arena of Valor/Honor of King (mobile games) has become a formal competition in WCG, and Mobile Legends have become a competition in CPL. Additionally, virtual reality (VR), augmented reality (AR), and optical motion capture technologies have undergone significant advancements and have been successfully integrated into the realm of video games. This integration has demonstrated their potential to expand and enhance the esports industry. Furthermore, the emergence of events such as the Formula One Esports Series and the inclusion of games like Just Dance in formal esports competitions have provided glimpses into the potential and future trends of the industry. Therefore, there is no doubt that esports is closely intertwined with the advancement of technology. Currently, AR and VR have not fully realized their expected outcomes, making it challenging to determine how esports will be presented in the future within a short period. As a result, the form of esports cannot be solely confined to games that rely on one specific technology or device at this stage, and there will be numerous possibilities within the esports industry.

###### 3.2.2.1.4 Participation

A total of 118 articles attempted to define esports from two perspectives based on the form of how people participate in esports: individually or in a team, remotely or face to face. Six out of 74 articles that discussed “participation” in the definition indicate that esports is a team-based activity, while the other indicated that esports could either be played individually or as a team. Overall, the majority of studies have concluded that esports involve competition between individuals, regardless of whether they participate as individuals or teams.

Furthermore, among the other 45 articles, 8 articles suggested that esports should be played within a local area network, with players gathering in stadiums and arenas, while 2 articles mentioned that esports could be played online or remotely. Regarding the usage of the term “online” in the definition, we referred to the literal meaning explanation provided by the Oxford Dictionary. According to the Oxford Dictionary, “online” means “controlled by or connected to a computer or the Internet”. This implies that the term “online” not only refers to playing esports on the Internet (remotely) but also includes playing within a local network. Based on the Oxford Dictionary, “online” refers to being “connected to another computer or connected to the internet or World Wide Web” [63]. Therefore, “online” not only suggests playing esports remotely but also in a local network or head-to-head. However, the reasons why esports should be head-to-head games or why they must be played across long distances have not been clarified in the article [33]. In fact, distance does not affect the content, form, or public recognition of esports. Therefore, in this article, distance will not be considered an attribute of esports. However, it is undeniable that since “online” refers to being networked to another computer, its underlying meaning suggests it is a dual-player or multiplayer activity, whether the players are in a cooperative or competitive relationship, once again indicating that esports should occur between people.

###### 3.2.2.1.5 Level

In this category, it is found that many codes conflict with each other. Out of 74 articles, 52 indicated that esports should be professional. “Professional”, on the one hand, means the skill level is high. On the other hand, it means making a living with esports or playing esports as a job or career. In the category of “goal”, we can also find that some researchers think the goal of esports should be to make a living with it, which also suggests the same meaning as “professional”. However, in 14 articles, it was demonstrated that esports could also be at an entertaining or semi-professional level. Meanwhile, many of the reviewed articles came up with the concept of “professional esports” and “pro-esports” [[64], [65], [66], [67], [68], [69]], “non-professional esports” [65], “recreational esports/casual and enthusiastic esports/leisure esports/amateur esports” [68,70,71], “esports for all” [69], or “collegial esports” [31,[72], [73], [74], [75]]. If esports should be limited to “professional”, these concepts are meaningless or contradictory. However, if esports is considered as a formal sport, we can attempt to explain or address this issue by referring to similar situations in traditional sports. In traditional sports, we categorize sports into different types based on the purpose of the activity, the types of participants, and the level of sports. For example, we have school sports, professional sports, mass sports, and so on. Therefore, as one type of sports, esports can also be approached using a similar classification method. Under this classification framework, esports should have a broader definition that highlights its core attributes. More proposed attributes could be included in narrower definitions of esports, such as specific types of esports (e.g., professional esports), rather than a general definition that applies to various contexts. For example, attributes like “making a living with” or “coordinated by different leagues, tournaments, or clubs” are more suitable for defining professional esports. Within this classification approach, these proposed attributes of esports achieve compatibility to some extent.

###### 3.2.2.1.6 Skills or literacy

A total of 37 articles propose that esports players should utilize their mental and physical skills to win the game instead of winning by chance. These abilities and literacy typically encompass communication skills, decision-making abilities, reaction speed, and information and communication technology literacy, among others [[76], [77], [78], [79]]. From this perspective, it can also be seen that most researchers acknowledge that physical skills and abilities are not only essential in traditional sports but also crucial in esports. In fact, they can be one of the key factors in achieving victory in esports. However, it cannot be ignored that card games (which are also formal events in the Asian Games and are also accepted as a conventional esports game by the community) do not depend on physical skills. Body movement exists in card games but can hardly be said to contribute to winning. Also, four articles used “cooperation” or “team spirit” in their definition, which also neglected individual esports. In sum, in this article, we do not take these categories to be essential features of esports.

###### 3.2.2.1.7 Sportification

Fifty-five articles considered esports as a sport directly in their definition. In this section, we also counted all the reviewed articles that discussed the relationship between sports and esports and obtained a proportion of the views that support esports as an official sport and those that do not. In the searched articles, a total of 50 papers clearly stated their attitude towards the issue. Only six papers held that esports is not a sport. Additionally, from the reviewed articles, it was found that Brazil, Bulgaria, China, Italy, Korea, Russia, and the US have already considered esports as a sport [80], and it is already under consideration in about 40 countries [81]. In this article, we accept esports as a sport.

###### 3.2.2.1.8 Emerging

Seven articles proposed that esports is an emerging and new phenomenon, and these articles were primarily published in 2020 [67,[82], [83], [84], [85], [86], [87]]. However, “emerging” is a relative view. In this article, we try to find a more objective and adaptable definition of esports. It is not certain, whether at this moment or in the near future, that “emerging” still fits/will fit the contemporary definition of esports. Although the form of esports is undergoing significant innovation, the concept and events of esports have existed for many years. The first widely-recognized concept of esports emerged in 2006 [25], and the first “Warcraft” tournament, which is conventionally recognized as an esports event, took place in 2002. Additionally, the inaugural WCG was held in 2000, which is one of the most significant major esports competitions worldwide. Therefore, we consider that esports has been an established activity for many years and is not solely a completely new and emerging phenomenon, and “emerging” will not be considered as a criterion for defining esports in this study.

##### 3.2.2.2 External environment or relationship

###### 3.2.2.2.1 Organization and commercial

Out of 299 articles, 120 mentioned “organized” in the definition of esports, and nine considered that esports should be “commercial”. These articles try to define esports from its external environment instead of the sports itself. They reckon there should be professional esports leagues and tournaments and social organizations or a governing body in esports, just like modern traditional professional competitive sports. In fact, this expression is more similar to the concept of professional sports. Likewise, this attribute will fit better into the definition of professional esports than general esports in this article.

From another perspective, during this period when esports is not yet universally recognized, when a certain category of esports games attains a significant player base, its level of commercialization steadily increases. This allows the general public to have opportunities to understand and become aware of it, and the game to gain acceptance and support within the esports community. This might indirectly contribute to the portrayal of esports as a commercialized and professional activity. This approach can also assist us in systematically studying and promoting esports as a formal sport.

###### 3.2.2.2.2 Audience and media

A total of 49 articles discussed media and audience when defining esports. This discussion did keep in line with the contemporary context and reflects the particularity of esports, a new type of sports in this age. For one thing, from a traditional perspective, being watched by an audience or being broadcast on TV or online is one of the attributes of professional sports. However, in esports, not only professional esports competitions are broadcast, but also casual competitions (not so organized as formal tournaments) or esports games or competitions at an entertaining level. Additionally, players include professional esports athletes or players at different levels who can also make a living through broadcasting [88,89]. They do not earn money like professional esports players (win prize money from tournaments or get paid by the clubs or institutions they are members of). Moreover, such content relies more on live streaming platforms rather than traditional media like traditional sports. These streamers can sometimes also bring the audience an experience of professional-level competitions, while others might only intend to entertain the audience with esports when live streaming. They usually get paid from live streaming platforms, audiences, and commercial sponsors [70,90]. This phenomenon rarely happens in traditional sports – it only exists in esports. Esports streamers usually do not play the games to entertain themselves like normal esports players, but most of them are usually skilled and experienced players or retired professional esports players, and the competitions they are involved in are watched by audiences as well [91]. Therefore, live streaming esports will be a separate type of esports in this article, different from mass esports and professional esports.

###### 3.2.2.2.3 Culture and industry

In a minority of articles, researchers are inclined to define esports in a broader sense, considering it as a culture or industry. Just like the relationship between sports and culture, there is no doubt that esports can be regarded as a cultural phenomenon or closely related to culture, but in this article, we, first of all, try to discuss esports in a narrower sense as a physical and practical activity (as the majority of the reviewed articles did), to distinguish esports from other computer games or activities, and determine its attributes in this age.

###### 3.2.2.2.4 Acceptance

As the terms and definitions of esports have been controversial, and the theoretical achievements are far behind the practical development of the esports industry, some scholars have proposed that the acceptance of the public and the media can be used as an important indicator to determine whether the activity or game can be regarded as an esports [23]. At the current stage, this is a feasible method in line with the contemporary situation of esports, but whether this can be regarded as a feature of esports or included in the definition of esports requires further theoretical explanation and discussion.

#### 3.2.3 Antecedents

In concept analysis, the antecedents refer to the event or condition that precedes the concept [24]. Based on the attributes of esports, its main antecedents are:

(i) competitive computer games/video games are involved
(ii) it occurs between people
(iii) it is facilitated by information and communication technology
(iv) physical or mental skills are required to compete with opponents or to vie for superiority

#### 3.2.4 Consequences

Consequences in concept analysis are the events and incidents that result from the concept [24]. Since nowadays esports is in a period of fast development and it is not yet well studied theoretically, the consequences of esports, for now, still need more evidence and research to be clarified. Some scholars have tried to explore the effects of esports on humans, including its possible positive and negative effects:

(i) esports can lead to the development of perceptual, attentional, cognitive, and social skills, the ability to manage tasks or information, self-regulated learning, and mental flexibility [76,[92], [93], [94], [95]];
(ii) some esports can enhance the motivation of traditional sports [96,97];
(iii) excessive play leads to some physical issues such as eye fatigue, blurry vision, lower back pain, and tension headaches [7,98];
(iv) excessive play results in a variety of psychological problems, including depression, anxiety, tension, sleep disturbance, addiction, and aggressive affect and behaviors [7,98,99].

## 4 Discussion

To provide more clarity on the variations of the concept of esports, based on the above results that different attributes belong to esports at different levels, we applied Sartori's “ladder of abstraction” to interpret the concept [100].

### 4.1 Ladder of abstraction

Sartori came up with two indicators of the concept: (i) the concept's “extension” – the range of cases that the concept can be applied in, and (ii) the concept's “intension” – the number of attributes that the concept has [100]. These two indicators are negatively correlated. The higher degree of intension a concept is, the more limited its application is. Conversely, the more limited attributes a concept has, the wider its application is [100].

**Fig. 4:**
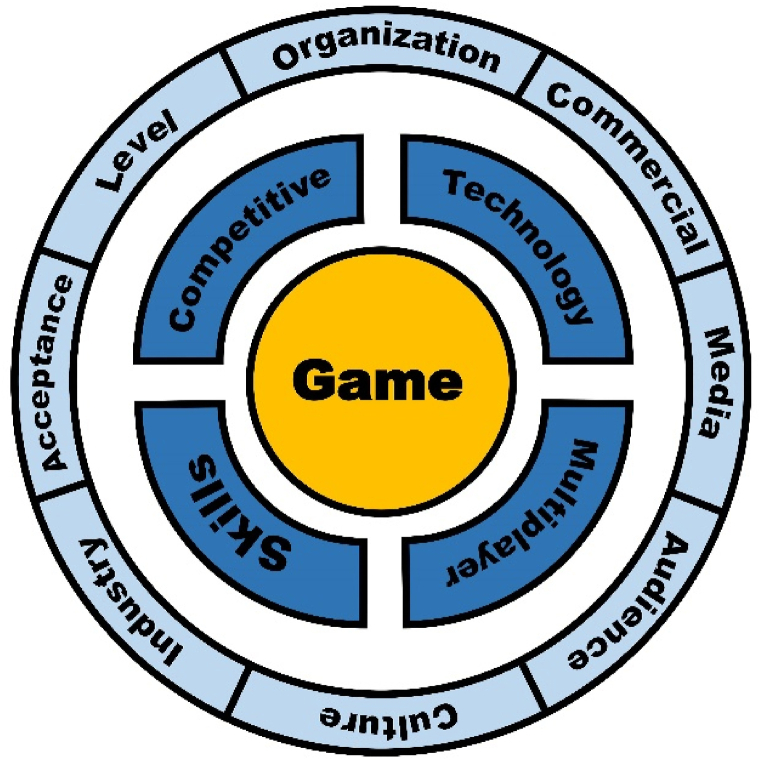
A framework of integrated esports attributes.

Based on reviewed articles, with a higher degree of intension, the definition of esports in a variety of articles tends to be professional esports. Many of the proposed attributes that are often associated with esports definition, such as high level, organization, career, and audience, mostly point towards a professional form of esports. However, there must also be a “root definition” that captures the core attribute, which can be applied in a wider range of cases and travel through time and context [101]. Therefore, a “root definition” of esports can also be extracted from the commonalities and the most fundamental attributes of different types of esports, which can be applied in most contexts when referring to esports. The fundamental and core characteristics of esports, as derived from these definitions, lie in the competitiveness, the application of physical and mental skills, the use of communication and information technology, and the interaction between individuals. These characteristics have also achieved a high level of recognition and consensus across different definitions. On the other hand, some other features reflect the esports of a higher degree of intension and have a narrower applicability. Fig. 4 integrates these characteristics and illustrates their hierarchical relationship. Specifically, live streaming esports, professional esports, and mass esports will be concepts of a higher degree of intension. Additionally, Table 4 illustrates the relationship between these attributes and these different categories of esports.

**Table 4:** Comparison of different categories of esports.

		Esports (core)	Live streaming esports	Mass esports	Professional esports
Computer games/video games		✓	✓	✓	✓
Competitive		✓	✓	✓	✓
Technology (facilitated by electronic systems)		✓	✓	✓	✓
Multiplayer		✓	✓	✓	✓
Skill and literacy (mental or physical abilities)		✓	✓	✓	✓
Level	Professional				✓
	Entertaining			✓	
Goal	To make a living				✓
	To entertain			✓	
Organized (coordinated by leagues and tournaments)					✓
Audience and media			✓		✓
Commercial			✓		✓
aAcceptance (by public and media)		✓	✓	✓	✓

a Acceptance will be considered as a method to determine whether it qualifies as an esports; however, there is a lack of theoretical evidence to support it as a core attribute of esports.

Table 5 shows the comparison between esports and other related concepts under the criterion. Exergames and active video games, for now, have not reached a professional level and have not been coordinated by different leagues and tournaments. Moreover, not all the exergames or active video games have to be competitive, and the goal of these kinds of games is more to entertain the players or for physical exercise, not to compete with opponents. From this perspective, it is more precise to conclude that exergames or active video games can be regarded as a new form of computer games or video games with higher intensity and more involved physical movement, which are, to some degree, different from current prevailing esports. Although some virtual sports have already appeared in professional esports competitions, in terms of definition, we can see that only when the virtual sports reach certain standards of esports (competitive, multiplayer, etc.) will it be included in esports. The emergence of the concept of “physical esports” appears to indicate the situation [[42], [43], [44]].

**Table 5:** Comparison between esports and related concepts.

		Computer games/video games	Esports	Physical esports	Virtual Sports	Exergame/active video games
Computer games/video games		✓	✓	✓	**√**_**1**_	✓
Competitive			✓	✓		
Technology		✓	✓	✓	✓	✓
Multiplayer			✓	✓		
Skill and literacy			✓	√_**2**_	√_**2**_	√_**2**_
Level						×
Goal	Make a living					×
	Entertain					
	Physical exercise					✓
Organized						×
Audience and media						×
Commercial						×
Acceptance			✓	✓		

1: The content of the games tends to simulate traditional sports.

2: The games place greater emphasis on the role of whole-body movement within the game, utilizing optical motion capture, VR, and AR technology.

“ × “: At this stage, there are currently no professional players, organizations, competitions, or live streaming platforms specifically dedicated to exergames or active video games.

In sum, this review will serve as a comprehensive summary of past discussions on esports. The framework and synthesis of existing definitions of esports presented in this paper will provide a reference standard for future research on esports-related topics. By identifying specific characteristics and themes from different definitions of esports, it helps us better differentiate esports from other activities, such as distinguishing esports players from general video game players. For example, the outlined criteria could further elaborate on why role-playing games should not be classified as esports. In role-playing games, the primary objective is not to defeat opponents or achieve victory. Instead, character development and immersive experiences in a virtual world are the key appeals of role-playing games, and it can also manifest as interaction between individuals and the virtual world instead of between people [7,102]. Furthermore, there are currently no esports events or organizations specifically dedicated to role-playing games. These attributes do not align with the core characteristics we have deduced from the definition of esports.

Additionally, this article also preliminarily explored the reasons why there is no widely recognized definition for esports in the current environment:

Firstly, it is because there has been a significant growth in esports-related research only since 2020 [4,5]. The influx of researchers in the esports field within a short period may lead to differences in understanding and perspectives on esports. We also call for future scholars interested in esports to base their research on standardized criteria for defining and categorizing esports-related research subjects.

Secondly, there are conflicting and controversial points within current definitions of esports, and resolving these disputes still requires more theoretical and empirical support. Overall, these contradictions mainly revolve around the following points: 1) Whether only professional esports can be considered esports, specifically encompassing aspects such as the presence of live streaming, audience and sponsorship, and the participation of highly skilled esports athletes. Furthermore, whether various other levels or scales of esports gaming activities or those with different purposes can be considered as esports. 2) Due to the different times when definitions came up, the forms of esports have undergone significant changes. In some definitions, esports is limited to computer games, but in the current industry, various forms of esports are recognized, including mobile games, motion-sensing games, and more [58,[103], [104], [105]]. 3) With advancements in technology, the traditional keyboard and mouse input method is no longer the only option. Full-body movement has also become one of the ways to interact with games, placing new demands on the skills and abilities of esports players. 4) Whether esports are limited to online or offline LAN-based competitions.

In this study, we also attempted to classify esports by considering the different characteristics of esports, making these existing divergent points in esports compatible within different categories of esports. This classification can also aid us in a systematic examination and advancement of esports as an established sport.

Thirdly, the form of esports is constantly evolving with technological advancements, and we are currently in a period of thriving growth in gaming and its associated technologies. This adds to the challenge of providing a comprehensive and objective definition of esports within a short timeframe. In addition to the divergences reflected in existing definitions, we can also anticipate the possibility of more divergences in the near future. For instance, Artificial Intelligence (AI) has been playing a significant role in games. We can see an increasing number of studies suggesting that AI technology is capable of creating opponents that are on par with human abilities [[106], [107], [108]]. Although currently, we recognize esports as competitions that occur between humans, once AI reaches a sufficient level of maturity, whether competitions between humans and AI can be regarded as esports will become an immediate and foreseeable controversy, similar to what we see in some existing intellectual sports [[109], [110], [111]].

Moreover, the subdivision and clarification of similar concepts in this study can help our future research study games or different types of esports more systematically. It also aids in understanding the current trends in the development of these games. For instance, distinctions between virtual sports and esports have been observed, but within the current esports industry, there are also commonalities and integration, such as the inclusion of Just Dance in international esports events. Additionally, establishing standardized terminology in the gaming field can avoid misuse in various studies and prevent misunderstandings of different research outcomes. Currently, there is a limited number of theoretical analyses on terms such as virtual sports and digital sports in the relevant literature, but as the popularity of these activities increases, especially with the International Olympic Committee's focus on virtual sports [112,113], the standardization of these terms will receive more attention. With the continuous development and popularization of the gaming industry, in future game research, we need to establish a standardized system of gaming terminology to standardize and promote these studies and help the public better understand and interpret the research findings.

Due to the limitation of searching only English literature in this paper, some discussions and research on esports from countries with well-developed esports industries and fields, such as China and South Korea, may be missing, resulting in gaps in a comprehensive understanding of esports. For example, there is the varying recognition and status of mobile games in the field of esports in Asian and Western countries [23]. In future research, it would be beneficial to include discussions from these countries that have more comprehensive developments in esports for a global perspective.

### 4.2 Limitations

The limitations of this paper include: (1) Only papers in English were searched and analyzed, and some of the research from countries with well-developed esports industries may not have been included due to language; (2) Due to the controversies and lack of consensus surrounding esports, as well as its relatively new nature compared to the development of traditional sports, there remains a dearth of comprehensive theoretical and empirical evidence supporting the various viewpoints on esports. In this paper, we have focused on exploring specific issues related to the development of esports by drawing comparisons to traditional sports in terms of theory and industry development. As future research on esports becomes more comprehensive and systematic, these issues can be further investigated and discussed more thoroughly; (3) Esports and the technology involved in it are experiencing rapid development, and its structure and industry are also in a transition period towards specialization in which it is hard to predict a complete and accurate future form of esports.

## 5 Conclusion

This article systematically collected and organized the adopted and accepted definitions in the field of esports. Through thematic coding, the attributes reflected in these definitions are extracted, and their commonalities and consensus are summarized. We proposed the essence of esports that can be applicable to a wide range of scenarios. In addition, we also identified specific contradictions and disagreements present in various definitions, which currently lack sufficient theoretical and practical evidence to resolve. By analyzing these discrepancies, we offered a preliminary explanation for the lack of consensus in the current understanding of esports. Drawing on theories and methodologies from traditional sports, we further proposed a classification of esports based on the purpose, participants, and skill level. Within this framework, certain contradictory themes or features achieve a certain level of compatibility. While we do not currently propose the concept of “school esports” similar to the one in traditional sports, there is a growing number of attempts to actively apply esports in education and universities, which are receiving increasing attention. Although these endeavors are still in their early stages, they show the potential. Additionally, under the guidelines of conceptual analysis, we analyzed alternative terms and related concepts of esports, and using the proposed esports framework, we identified commonalities and differences among these activities. We also observe overlapping areas or “gray zones” between these activities, resulting from the development of the gaming and esports industry, as well as related gaming technologies. Some of these activities are undergoing convergence.

It is important to acknowledge the dynamic nature of concepts. With advancements in the esports industry and technology, particularly in motion capture, AR, and VR, the scale and form of esports continue to expand. Consequently, in the near future, the understanding and definition of esports may require further refinement. Furthermore, while the majority of research and an increasing number of countries classify esports as a formal sport, there is also a need for further transparency and clarity regarding the definition of modern sports in this era.

The framework and synthesis of established esports definitions presented in this paper can serve as a valuable reference for future research in the field. Moreover, the subdivision and clarification of related concepts contribute to a more systematic approach to studying games and different forms of esports. With the ongoing growth and development of the gaming industry and related scientific research, it becomes essential to establish a standardized system of gaming terminology to promote and standardize research, as well as enhance public understanding and interpretation of research findings.

## Funding statement

This study has received no funding.

## Data availability statement

Data included in article/supp. material/referenced in article. The complete raw dataset will be provided upon request.

## CRediT authorship contribution statement

**Di Tang:** Writing – review & editing, Writing – original draft, Methodology, Formal analysis, Data curation, Conceptualization. **Raymond Kim-wai Sum:** Writing – review & editing, Supervision, Project administration. **Minghui Li:** Methodology, Conceptualization. **Ruisi Ma:** Methodology, Formal analysis. **Peichi Chung:** Writing – review & editing, Supervision. **Ray Wai-keung Ho:** Formal analysis, Data curation.

## Declaration of competing interest

The authors declare that they have no known competing financial interests or personal relationships that could have appeared to influence the work reported in this paper.

## References

[1] Block S., Haack F. (2021). SHS Web of Conferences.

[2] Hamari J., Sjöblom M. (2017). What is eSports and why do people watch it?. Internet Res..

[3] Reitman J.G. (2019). Esports research: a literature review. Game. Cult..

[4] Jeong D., Youk S. (2023). Refining esports: a quantitative cartography of esports literature. Entertainment Computing.

[5] Chiu W. (2021). Knowledge mapping and sustainable development of esports research: a bibliometric and visualized analysis. Sustainability.

[6] Parshakov P., Coates D., Zavertiaeva M. (2018). Is diversity good or bad? Evidence from eSports teams analysis. Appl. Econ..

[7] Chan G. (2022). The impact of eSports and online video gaming on lifestyle behaviours in youth: a systematic review. Comput. Hum. Behav..

[8] Dongsheng Y., Xiaohang Y., Daofeng K. (2011). 2011 International Conference on Future Computer Science and Education, ICFCSE 2011.

[9] Karsenti T., Arai K., Bhatia R., Kapoor S. (2020). 4th Future Technologies Conference, FTC 2019.

[10] Madden D., Harteveld C. (2021). Proceedings of the 2021 CHI Conference on Human Factors in Computing Systems.

[11] Atalay A., Topuz A.C. (2018). What is being played in the world? Mobile esport applications. Universal Journal of Educational Research.

[12] Funk D.C., Pizzo A.D., Baker B.J. (2021). eSport management: embracing eSport education and research opportunities. Sport Manag. Rev..

[13] Hemphill D. (2005). Cybersport. Journal of the Philosophy of Sport.

[14] van Hilvoorde I., Pot N. (2016). Embodiment and fundamental motor skills in eSports. Sport Ethics Philos..

[15] Rejikumar G. (2022). Towards a theory of well-being in digital sports viewing behavior. J. Serv. Market..

[16] Peters M.D. (2022). Best practice guidance and reporting items for the development of scoping review protocols. JBI evidence synthesis.

[17] Sabiston C.M. (2022). Scoping reviews and rapid reviews. Int. Rev. Sport Exerc. Psychol..

[18] Arksey H., O'Malley L. (2005). Scoping studies: towards a methodological framework. Int. J. Soc. Res. Methodol..

[19] Tricco A.C. (2018). PRISMA extension for scoping reviews (PRISMA-ScR): checklist and explanation. Ann. Intern. Med..

[20] Gibbs G. (2018).

[21] Kassarjian H.H. (1977). Content analysis in consumer research. J. Consum. Res..

[22] Rust R.T., Cooil B. (1994). Reliability measures for qualitative data: theory and implications. J. Market. Res..

[23] Taylor T.L. (2020). The rise of massive multiplayer online games, esports, and game live streaming an interview with T. L.Taylor. AMERICAN JOURNAL OF PLAY.

[24] Rodgers B.L., Knafl K.A. (2000).

[25] Wagner M.G. (2006). International Conference on Internet Computing.

[26] Wagner M. (2007).

[27] Freeman G., Wohn D.Y. (2017). Proceedings of the 2017 CHI Conference Extended Abstracts on Human Factors in Computing Systems.

[28] Witkowski E. (2012). On the digital playing field. Game. Cult..

[29] Pedraza-Ramirez I. (2020). Setting the scientific stage for esports psychology: a systematic review. Int. Rev. Sport Exerc. Psychol..

[30] Hutchins B. (2008). Signs of meta-change in second modernity: the growth of e-sport and the World Cyber Games. New Media Soc..

[31] Jenny S.E. (2016). Virtual(ly) athletes: where eSports fit within the definition of “sport”. Quest.

[32] (2017). The associated press., esports or eSports?. https://www.dexerto.com/news/esports-esports-associated-press928puts-end-popular-debate/3223.

[33] Scholz T.M. (2019).

[34] Kim J.S., Kim M.G. (2012). SIGGRAPH Asia 2012 Posters*, SA* 2012.

[35] Kim J.S., Kim M.G. (2012). 2012 International Conference on ICT Convergence: ""Global Open Innovation Summit for Smart ICT Convergence"", ICTC 2012.

[36] Oh Y., Yang S. (2010). Defining exergames & exergaming. Proceedings of meaningful play.

[37] Mears D., Hansen L. (2009). Active gaming: definitions, options and implementation. Article# 5 in a 6-part series. Strategies.

[38] del Castillo H. (2011). 2011 4TH INTERNATIONAL CONFERENCE OF EDUCATION.

[39] Hebbel-Seeger A. (2012). The relationship between real sports and digital adaptation in e-sport gaming. Int. J. Sports Mark. Spons..

[40] Kamijo K.i. (1993). In Proc. HCI International'93.

[41] Morizono T., Kurahashi K., Kawamura S. (1997). Proceedings of International Conference on Robotics and Automation.

[42] Kriglstein S. (2021). Extended Abstracts of the 2021 CHI Conference on Human Factors in Computing Systems.

[43] Yasumoto M., Shida K., Teraoka T., Marcus A., Rosenzweig E. (2020). 9th International Conference on Design, User Experience, and Usability, DUXU 2020, Held as Part of the 22nd International Conference on Human-Computer Interaction, HCII 2020.

[44] Yasumoto M., Teraoka T. (2019). SIGGRAPH Asia 2019 XR - International Conference on Computer Graphics and Interactive Techniques, SA 2019.

[45] Martončik M. (2015). e-Sports: playing just for fun or playing to satisfy life goals?. Comput. Hum. Behav..

[46] Taylor N. (2016). Play to the camera: video ethnography, spectatorship, and e-sports. Convergence.

[47] Juul J. (2010). The game, the player, the world: looking for a heart of gameness. Plurais Revista Multidisciplinar.

[48] Kelley D. (2013).

[49] Suits B. (1988). Tricky triad: games, play, and sport. J. Philos. Sport.

[50] Tekinbas K.S., Zimmerman E. (2003).

[51] Rothwell G., Shaffer M. (2019). eSports in K-12 and Post-Secondary schools. Educ. Sci..

[52] Scott M.J. (2021).

[53] Amazan-Hall K. (2018). Diversity and inclusion in esports programs in higher education: leading by example at UCI. Int. J. Gaming Comput.-Mediated Simulat. (IJGCMS).

[54] Bickmann P. (2021). Comparison of reaction time between esports players of different genres and sportsmen. International Journal of eSports Research (IJER).

[55] Bonnar D. (2019). Risk factors and sleep intervention considerations in esports: a review and practical guide. Sleep Medicine Research.

[56] Tregel T., Fletcher B. (2021). 7th Joint International Conference on Serious Games, JCSG 2021.

[57] Bornemark O. (2013). Umeå’s 16th Student Conference in Computing Science.

[58] Asian Electronic Sports Federation https://www.aesf.com/news-media.

[59] Jang W.W., Byon K.K. (2020). Antecedents of esports gameplay intention: genre as a moderator. Comput. Hum. Behav..

[60] Trotter M.G. (2022).

[61] Conroy E. (2021). Boosting: rank and skill deception in esports. Entertainment Computing.

[62] Iwatsuki T., Hagiwara G., Dugan M.E. (2022). Effectively optimizing esports performance through movement science principles. Int. J. Sports Sci. Coach..

[63] Oxford dictionary, meaning of “online” in UK English. https://www.lexico.com/definition/online.

[64] Happonen A., Minashkina D. (2019).

[65] Harris B., Igelman A.A., Pollock M. (2020). peer-to-peer esports sanctioning organization. Gaming Law Rev..

[66] Ke X., Wagner C. (2020). Global pandemic compels sport to move to esports: understanding from brand extension perspective. Managing Sport and Leisure.

[67] Sainz I., Collado-Mateo D., Del Coso J. (2020). Effect of acute caffeine intake on hit accuracy and reaction time in professional e-sports players. Physiol. Behav..

[68] Seo Y. (2016). Professionalized consumption and identity transformations in the field of eSports. J. Bus. Res..

[69] Kee-Young Y. (2020). Policies and legislation of e-sports in korea. International Sports Law Review Pandektis.

[70] Jang W.W. (2021). Mediating effect of esports content live streaming in the relationship between esports recreational gameplay and esports event broadcast. Sport Bus. Manag.: Int. J..

[71] Zimmer R.T. (2022). Acute effects of esports on the cardiovascular system and energy expenditure in amateur esports players. Front Sports Act Living.

[72] Cho A., Tsaasan A.M., Steinkuehler C. (2019). 14th International Conference on the Foundations of Digital Games, FDG 2019.

[73] Taylor N. (2020). The numbers game: collegiate esports and the instrumentation of movement performance. Sports, Society, and Technology: Bodies, Practices, and Knowledge Production.

[74] Kauweloa N.S., Winter J.S. (2019). 52nd Annual Hawaii International Conference on System Sciences, HICSS 2019.

[75] Murray J., Pricce E.B., Decker S. (2021). The mission value of collegiate esports. Sport J..

[76] Zhong Y. (2022).

[77] Sörman D.E. (2022). Relationships between Dota 2 expertise and decision-making ability. PLoS One.

[78] Koposov D. (2020). 29th IEEE International Symposium on Industrial Electronics, ISIE 2020.

[79] Nagorsky E., Wiemeyer J. (2020). The structure of performance and training in esports. PLoS One.

[80] Weiss T. (2008). https://aisel.aisnet.org/digit2008/7/.

[81] International eSports Federation Member nations. http://www.ie-sf.org/about/#member-nations.

[82] Yang A., Liu D., Santhanam R., Garimella A. (2021). 19th Workshop on E-Business, WeB 2020.

[83] Lee S. (2020). Sleep characteristics and risk factors of Korean esports athletes: an exploratory study. Sleep Medicine Research.

[84] Wanyi T. (2018). Understanding esports from the perspective of team dynamics. Sport J..

[85] Railsback D., Caporusso N., Ahram T.Z. (2019). AHFE 2018 International Conferences on Human Factors and Wearable Technologies, and Human Factors in Game Design and Virtual Environments, 2018.

[86] Freeman G., Wohn D.Y. (2017). CHI PLAY'17: PROCEEDINGS OF THE ANNUAL SYMPOSIUM ON COMPUTER-HUMAN INTERACTION IN PLAY.

[87] Wang H., Huo H., Zhang D. (2021). Structural analysis of e-sports industrial association network based on complex network theory. Rev. Psicol. Deporte.

[88] Grayson N. (2019).

[89] Taylor H. (2018).

[90] Wohn D.Y., Freeman G. (2019). Live streaming, playing, and money spending behaviors in eSports. Game. Cult..

[91] Xu Q., Kim H., Billings A.C. (2022). Let's watch live streaming: how streamer credibility influences brand attitude in esports streamer marketing. Commun. Sport.

[92] Bediou B. (2018). Meta-analysis of action video game impact on perceptual, attentional, and cognitive skills. Psychol. Bull..

[93] Bisht A., Sharma H.K., Choudhury T., Sharma D.K. (2022). Effect of Esports Among Students in COVID Era.

[94] Martynenko S.E. (2021).

[95] Palaus M. (2017). Neural basis of video gaming: a systematic review. Frontiers in human neuroscience.

[96] Kane D. (2020). An investigation to determine if sport video games helps community college students become interested in real-life sports. Sport J..

[97] Tang D. (2023). Beyond the screen: do esports participants really have more physical health problems?. Sustainability.

[98] Palanichamy T. (2020). Influence of Esports on stress: a systematic review. Ind Psychiatry J.

[99] Choi C., Ma Hums, Bum C.H. (2018). Impact of the family environment on juvenile mental health: eSports online game addiction and delinquency. Int J Environ Res Public Health.

[100] Sartori G. (1970). Concept misformation in comparative politics. Am. Polit. Sci. Rev..

[101] Collier D., Levitsky S. (1997). Democracy with adjectives: conceptual innovation in comparative research. World Polit..

[102] Bostan B. (2009). Requirements analysis of presence: insights from a RPG game. Computers in Entertainment (CIE).

[103] Yang Z. (2022). Interpretable real-time win prediction for honor of kings—a popular mobile MOBA esport. IEEE Transactions on Games.

[104] Parry J., Giesbrecht J. (2023). Esports, real sports and the olympic virtual series. J. Philos. Sport.

[105] Yingji L. (2021). 2021 International Conference on Information Technology and Contemporary Sports (TCS).

[106] Vinyals O. (2019). Grandmaster level in StarCraft II using multi-agent reinforcement learning. Nature.

[107] Ling B. (2022). International Conference on Neural Computing for Advanced Applications.

[108] Berner C. (2019).

[109] Wimmer P. (2019). Seminar Report: Artificial Intelligence for Games SS19.

[110] Xu C. (2022). 2022 IEEE 22nd International Conference on Software Quality, Reliability, and Security Companion (QRS-C).

[111] Risi S., Preuss M. (2020). From chess and atari to starcraft and beyond: how game ai is driving the world of ai. KI-Künstliche Intelligenz.

[112] Li Z., Yang Y. (2022). Of Papers Presented at 2022 3rd Asia Sport Science Conference.

[113] Lu Z. (2022). Forging a link between competitive gaming, sport and the Olympics: history and new developments. Int. J. Hist. Sport.

