# Supplementary material for: What is esports? A systematic scoping review and concept analysis of esports
Source: Heliyon. 2023 Dec 3;9(12):e23248. doi: 10.1016/j.heliyon.2023.e23248 (PMC10750068; doi:10.1016/j.heliyon.2023.e23248)
Supplement: Multimedia component 2 [file mmc2.docx]

**Appendix B**

**The List of Included Literature**

Abbasi, A. Z., Asif, M., Hollebeek, L. D., Islam, J. U., Ting, D. H., & Rehman, U. (2021). The effects of consumer esports videogame engagement on consumption behaviors [Article]. Journal of Product and Brand Management, 30(8), 1194-1211. https://doi.org/10.1108/JPBM-04-2020-2839

Adamus, T. (2012). Playing computer games as electronic sport: In search of a theoretical framework for a new research field. In Computer Games and New Media Cultures: A Handbook of Digital Games Studies (pp. 477-490). Springer Netherlands. https://doi.org/10.1007/978-94-007-2777-9_30

Adinolf, S., & Türkay, S. (2018). Toxic behaviors in eSports games: Player perceptions and coping strategies. 5th ACM SIGCHI Annual Symposium on Computer-Human Interaction in Play, CHI PLAY 2018,

Alkier, R., & Demirkiran, K. P. (2019). ESPORTS: THE INVESTMENT OPPORTUNITY OF THE DECADE FOR REGIONAL TOURISM DEVELOPMENT 8TH INTERNATIONAL SCIENTIFIC SYMPOSIUM ECONOMY OF EASTERN CROATIA - VISION AND GROWTH,

AlMarzooqi, M. A., Alhaj, O. A., Alrasheed, M. M., Helmy, M., Trabelsi, K., Ebrahim, A., Hattab, S., Jahrami, H. A., & Ben Saad, H. (2022). Symptoms of Nomophobia, Psychological Aspects, Insomnia and Physical Activity: A Cross-Sectional Study of ESports Players in Saudi Arabia. Healthcare (Basel), 10(2). https://doi.org/10.3390/healthcare10020257

Almesalm, S., Bakhsh, N., & Khan, S. A. (2021). Applying an Iterative Design Approach to Kafu Games to Enhance the User Experience of Gamers in Saudi Arabia. In T. Z. Ahram & C. S. Falcao (Eds.), AHFE Conferences on Usability and User Experience, Human Factors and Wearable Technologies, Human Factors in Virtual Environments and Game Design, and Human Factors and Assistive Technology, 2021 (Vol. 275, pp. 767-773): Springer Science and Business Media Deutschland GmbH.

Anderson, C. G., Tsaasan, A. M., Reitman, J., Lee, J. S., Wu, M., Steel, H., Turner, T., & Steinkuehler, C. (2018). Understanding esports as a STEM career ready curriculum in the wild. 10th International Conference on Virtual Worlds and Games for Serious Applications, VS-Games 2018,

Anderson, S. L. (2017). Watching people is not a game: Interactive online corporeality, Twitch.tv and videogame streams [Article]. Game Studies, 17(1). https://www.scopus.com/inward/record.uri?eid=2-s2.0-85026839764&partnerID=40&md5=82b93b1f4012843c7eae76384e285f4a

Andre, T. L., Walsh, S. M., ValladÃo, S., & Cox, D. (2020). Physiological and Perceptual Response to a Live Collegiate Esports Tournament. Int J Exerc Sci, 13(6), 1418-1429.

Ani, R., Harikumar, V., Devan, A. K., & Deepa, O. S. (2019). Victory prediction in league of legends using feature selection and ensemble methods. 2019 International Conference on Intelligent Computing and Control Systems, ICCS 2019,

Argilés, M., Quevedo-Junyent, L., & Erickson, G. (2022). Topical Review: Optometric Considerations in Sports Versus E-Sports. Percept Mot Skills, 315125211073401. https://doi.org/10.1177/00315125211073401

Atalay, A., & Topuz, A. C. (2018). What is being played in the world? Mobile esport applications [Article]. Universal Journal of Educational Research, 6(6), 1243-1251. https://doi.org/10.13189/ujer.2018.060615

Baltezarević, B., & Baltezarević, V. (2019). eSPORTS AS A NEW PLAYGROUND. / eSPORT KAO NOVA IGRAONICA. Facta Universitatis: Series Physical Education & Sport, 17(1), 23-30. https://search.ebscohost.com/login.aspx?direct=true&db=s3h&AN=137869723&site=ehost-live&scope=site

Baltezarević, R., & Baltezarević, B. (2018). THE IMPACT OF VIDEO GAMES ON THE FORMATION OF eSPORTS. / UTICAJ VIDEO IGRE NA FORMIRANJE ESPORTA. Facta Universitatis: Series Physical Education & Sport, 16(1), 137-147. https://search.ebscohost.com/login.aspx?direct=true&db=s3h&AN=131285569&site=ehost-live&scope=site

Bányai, F., Griffiths, M. D., Király, O., & Demetrovics, Z. (2019). The psychology of esports: A systematic literature review. Journal of gambling studies, 35(2), 351-365.

Banyai, F., Griffiths, M. D., Demetrovics, Z., & Kiraly, O. (2019). The mediating effect of motivations between psychiatric distress and gaming disorder among esport garners and recreational gamers. Comprehensive Psychiatry, 94. https://doi.org/10.1016/j.comppsych.2019.152117

Bayrakdar, A., Yildiz, Y., & Bayraktar, I. (2020). Do e-athletes move? A study on physical activity level and body composition in elite e-sports. PHYSICAL EDUCATION OF STUDENTS, 24(5), 259-264. https://doi.org/10.15561/20755279.2020.0501

Behnke, M., Gross, J. J., & Kaczmarek, L. D. (2020). The role of emotions in esports performance. Emotion. https://doi.org/10.1037/emo0000903

Beres, N. A., Klarkowski, M., & Mandryk, R. L. (2021). Under Pressure: Exploring Choke and Clutch in Competitive Video Games [Conference Paper]. Proceedings of the ACM on Human-Computer Interaction, 5(CHIPLAY), Article 239. https://doi.org/10.1145/3474666

Bhatt, N. (2021). ESPORTS: From Why Do It to How Can I Get Into It? Parks & Recreation, 56(2), 46-51. https://search.ebscohost.com/login.aspx?direct=true&db=s3h&AN=148707704&site=ehost-live&scope=site

Bisht, A., Sharma, H. K., & Choudhury, T. (2022). Effect of esports Among Students in COVID Era. In D. K. Sharma, S. Peng, R. Sharma, & D. A. Zaitsev (Eds.), (Vol. 373, pp. 517-524): Springer Science and Business Media Deutschland GmbH.

Block, F., Hodge, V., Hobson, S., Sephton, N., Devlin, S., Ursu, M. F., Drachen, A., & Cowling, P. I. (2018). Narrative bytes: Data-driven content production in esports. 5th ACM International Conference on Interactive Experiences for TV and Online Video, TVX 2018,

Blom, P. M., Bakkes, S., & Spronck, P. (2019). Towards multi-modal stress response modelling in competitive league of legends. 2019 IEEE Conference on Games, CoG 2019,

Boffard, R. (2016). ESports: How To Make Money [Article]. Engineering and Technology, 11(4), 66-69. https://doi.org/10.1049/et.2016.0407

Boguslavskaya, V., Budnik, E., Azizulova, A., & Sharakhina, L. V. (2018). Cybersport community: Social structures transformation as a basis for intercultural dialogue. In S. S. Bodrunova (Ed.), 5th International Conference on Internet Science, INSCI 2018 (Vol. 11193 LNCS, pp. 300-311): Springer Verlag.

Bonnar, D., Castine, B., Kakoshcke, N., & Sharp, G. (2019). Sleep and performance in Eathletes: for the win! Sleep Health, 5(6), 647-650. https://doi.org/10.1016/j.sleh.2019.06.007

Bonnar, D., Lee, S., Gradisar, M., & Suh, S. (2019). Risk factors and sleep intervention considerations in esports: A review and practical guide [Article]. Sleep Medicine Research, 10(2), 59-66. https://doi.org/10.17241/smr.2019.00479

Bonnar, D., Lee, S., Roane, B. M., Blum, D. J., Kahn, M., Jang, E., Dunican, I. C., Gradisar, M., & Suh, S. (2022). Evaluation of a Brief Sleep Intervention Designed to Improve the Sleep, Mood, and Cognitive Performance of Esports Athletes. Int J Environ Res Public Health, 19(7). https://doi.org/10.3390/ijerph19074146

Bornemark, O. (2013, June). Success factors for e-sport games. In Proceedings of Umeå's 16th student conference in computing science: USCCS 2013 (pp. 1-12).

Borowy, M. (2013). Pioneering eSport: the experience economy and the marketing of early 1980s arcade gaming contests. International Journal of Communication, 7, 21.

Borowy, M., & Jin, D. Y. (2016). Mega-events of the future The experience economy, the Korean connection, and the growth of eSport (Vol. 52).

Bosc, G., Kaytoue, M., Raïssi, C., & Boulicaut, J. F. (2013). Strategic pattern discovery in RTS-games for E-sport with sequential pattern mining. 1st Workshop on Machine Learning and Data Mining for Sports Analytics, MLSA 2013,

Brevers, D., King, D. L., & Billieux, J. (2020). Delineating adaptive esports involvement from maladaptive a self. Current Opinion in Psychology, 36, 141-146. https://doi.org/10.1016/j.copsyc.2020.07.025

Burk, D. L. (2013). Owning e-Sports: Proprietary rights in professional computer gaming. University of Pennsylvania Law Review, 161(6), 1535-1578.

Buzzelli, A., & Draper, J. (2021). Are They Athletes? A Self-Assessment of Athletic Identity Measurement and Perceived Benefits of Collegiate Esports Participants. Recreational Sports Journal. https://doi.org/10.1177/15588661211033252

Campbell, M. J., Toth, A. J., Moran, A. P., Kowal, M., & Exton, C. (2018). eSports: A new window on neurocognitive expertise? In S. Marcora & M. Sarkar (Eds.), Progress in Brain Research (Vol. 240, pp. 161-174): Elsevier B.V.

Canavarro, G. G., Sequeiros, J. B. F., & Fernandes, F. (2019). Game design decisions and communication theories applied to esports: A literature review. In N. Zagalo, L. Costa, A. I. Veloso, & O. Mealha (Eds.), 11th International Conference on Videogame Sciences and Arts, VJ 2019 (Vol. 1164 CCIS, pp. 123-135): Springer Science and Business Media Deutschland GmbH.

Candela, J., & Jakee, K. (2018). Can ESports Unseat the Sports Industry? Some Preliminary Evidence from the United States. Choregia, 14(2), 55-71. https://search.ebscohost.com/login.aspx?direct=true&db=s3h&AN=134042823&site=ehost-live&scope=site

Carbonie, A., Guo, Z., & Cahalane, M. (2018). Positive personal development through eSports. 22nd Pacific Asia Conference on Information Systems - Opportunities and Challenges for the Digitized Society: Are We Ready?, PACIS 2018,

Cavadenti, O., Codocedo, V., Boulicaut, J. F., Kaytoue, M., & Ieee. (2016). What did I do Wrong in my MOBA Game?: Mining Patterns Discriminating Deviant Behaviours PROCEEDINGS OF 3RD IEEE/ACM INTERNATIONAL CONFERENCE ON DATA SCIENCE AND ADVANCED ANALYTICS, (DSAA 2016),

Cestino-Castilla, J., Macey, J., & McCauley, B. (2021). Aiming for validity: The experience of conflicts in legitimacy judgments in esports actors and new grassroots activism. 5th International GamiFIN Conference, GamiFIN 2021,

Chan, G., Huo, Y., Kelly, S., Leung, J., & Tisdale, C. (2022). The impact of eSports and online video gaming on lifestyle behaviours in youth: A systematic review [Article]. Computers in Human Behavior, 126, Article 106974. https://doi.org/10.1016/j.chb.2021.106974

Charleer, S., Gerling, K., Gutiérrez, F., Cauwenbergh, H., Luycx, B., & Verbert, K. (2018). Real-time dashboards to support esports spectating. 5th ACM SIGCHI Annual Symposium on Computer-Human Interaction in Play, CHI PLAY 2018,

Che, J. J. (2018). Study on Videogame Industry and Its General Developing Trend 2018 5TH INTERNATIONAL CONFERENCE ON BUSINESS, ECONOMICS AND MANAGEMENT (BUSEM 2018),

Chen, Y. Q., Mei, J., & Huang, S. (2020). Machine Learning and Data mining on the innovation of E-sports industry. INTERNATIONAL JOURNAL OF EDUCATION AND INFORMATION TECHNOLOGIES, 14, 121-132. https://doi.org/10.46300/9109.2020.14.15

Chikish, Y., Carreras, M., & García, J. (2019). eSports: A new era for the sports industry and a new impulse for the research in sports (and) economics. Sports (and) Economics, 477-508.

Chiu, W., Fan, T. C. M., Nam, S. B., & Sun, P. H. (2021). Knowledge mapping and sustainable development of esports research: A bibliometric and visualized analysis [Review]. Sustainability (Switzerland), 13(18), Article 10354. https://doi.org/10.3390/su131810354

Cho, A., Tsaasan, A. M., & Steinkuehler, C. (2019). The building blocks of an educational esports league: Lessons from year one in orange county high schools. 14th International Conference on the Foundations of Digital Games, FDG 2019,

Choi, C., Hums, M. A., & Bum, C. H. (2018). Impact of the Family Environment on Juvenile Mental Health: eSports Online Game Addiction and Delinquency. Int J Environ Res Public Health, 15(12). https://doi.org/10.3390/ijerph15122850

Choi, E. Y., & Choi, Y. S. (2017). An analysis of game piracy determinants in E-sports using the theory of reasoned action [Article]. Information (Japan), 20(5), 3439-3446. https://www.scopus.com/inward/record.uri?eid=2-s2.0-85035051670&partnerID=40&md5=68eda5f3ac8fcc924961c4c20ab3a884

Chulhwan, C. (2019). Understanding Media Consumption of Electronic Sports through Spectator Motivation, Using Three Different Segmentation Approaches: The Levels of Addiction, Passion, and Fan Identification. Sport Mont, 17(1), 3-8. https://search.ebscohost.com/login.aspx?direct=true&db=s3h&AN=134593828&site=ehost-live&scope=site

Ciloglu, F., Eroglu, Y., & Ikizler, H. C. (2020). 5W1H of HADO, From the Athlete's Perspective. AMBIENT SCIENCE, 7, 371-374. https://doi.org/10.21276/ambi.2020.07.sp1.ga07

Columb, D., Griffiths, M. D., & O'Gara, C. (2022). Online gaming and gaming disorder: more than just a trivial pursuit. Ir J Psychol Med, 39(1), 1-7. https://doi.org/10.1017/ipm.2019.31

Conroy, E., Toth, A. J., & Campbell, M. J. (2022). The effect of computer mouse mass on target acquisition performance among action video gamers. Appl Ergon, 99, 103637. https://doi.org/10.1016/j.apergo.2021.103637

Cortes, P., Huang, H., & Leung, A. (2021). Analysis of Legal Challenges Facing Esports in The People's Republic of China, Including The Macau SAR. GAMING LAW REVIEW-ECONOMICS REGULATION COMPLIANCE AND POLICY, 25(10), 414-425. https://doi.org/10.1089/glr2.2021.0012

Costa, R. S. M. (2020). Understanding the labor of professional eSports players. 7th ACM SIGCHI Annual Symposium on Computer-Human Interaction in Play, CHI PLAY 2020,

Cranmer, E. E., Han, D.-I. D., van Gisbergen, M., & Jung, T. (2021). Esports matrix: Structuring the esports research agenda. Computers in Human Behavior, 117. https://doi.org/10.1016/j.chb.2020.106671

Cumming, D. J. J., Gibbs, M., & Smith, W. (2021). Constructing Authentic Spectatorship at an Esports Bar [Article]. Journal of Contemporary Ethnography. https://doi.org/10.1177/08912416211031661

Cunningham, G. B., Fairley, S., Ferkins, L., Kerwin, S., Lock, D., Shaw, S., & Wicker, P. (2021). eSport: Construct specifications and implications for sport management. Sport Management Review, 21(1), 1-6. https://doi.org/10.1016/j.smr.2017.11.002

Curtin, A., Watson, J., Topoglu, Y., DeFilippis, N., Ye, H., Suri, R., & Ayaz, H. (2021). Interpersonal Synchrony Protocol for Cooperative Team Dynamics During Competitive E-Gaming. In H. Ayaz, U. Asgher, & L. Paletta (Eds.), AHFE Conferences on Neuroergonomics and Cognitive Engineering, Industrial Cognitive Ergonomics and Engineering Psychology, and Cognitive Computing and Internet of Things, 2021 (Vol. 259, pp. 149-156): Springer Science and Business Media Deutschland GmbH.

Darvin, L., Vooris, R., & Mahoney, T. (2020). The Playing Experiences of Esports Participants: An Analysis of Hostility and Treatment Discrimination in Esports Environments. Journal of Athlete Development & Experience (JADE), 2(1), 36-50. https://search.ebscohost.com/login.aspx?direct=true&db=s3h&AN=149390235&site=ehost-live&scope=site

DeArmond, M. C., Shelton, B. E., & Hsu, Y. C. (2022). The gap between Korean esports and educational gaming [Article]. International Journal of Game-Based Learning, 12(1). https://doi.org/10.4018/IJGBL.287828

Demediuk, S., Murrin, A., Bulger, D., Hitchens, M., Drachen, A., Raffe, W. L., & Tamassia, M. (2018). Player retention in league of legends: A study using survival analysis. 2018 Australasian Computer Science Week Multiconference, ACSW 2018,

Demediuk, S., York, P., Drachen, A., Walker, J. A., & Block, F. (2019). Role identification for accurate analysis in dota 2. 15th AAAI Conference on Artificial Intelligence and Interactive Digital Entertainment, AIIDE 2019,

Denoo, M., Bibert, N., & Zaman, B. (2021). Disentangling the motivational pathways of recreational esports gamblers: A laddering study. 10th International Conference on Materials Processing and Characterisation, ICMPC 2020,

Dipa Kalyani Sujata, P., Bianca Sardjono, S., & Putri Hendratno, S. (2022). Esport Ecosystem, Financial Behavior, and Carbon Emissions in Indonesian Urban Area.

Eichelbaum, J., Hänsch, R., & Hellwich, O. (2018). Classification of Icon Type and Cooldown State in Video Game Replays. In B. ter Haar Romeny, F. Karray, & A. Campilho (Eds.), 15th International Conference on Image Analysis and Recognition, ICIAR 2018 (Vol. 10882 LNCS, pp. 227-234): Springer Verlag.

Ekdahl, D., & Ravn, S. (2021). Social bodies in virtual worlds: Intercorporeality in Esports. Phenomenology and the Cognitive Sciences. https://doi.org/10.1007/s11097-021-09734-1

Erb, E. K., Dowdell, B., Glickman, E., Ridgel, A., & Barkley, J. (2021). The Effects Of Fortnite On Measures Of Cognition. Medicine & Science in Sports & Exercise, 53, 343-343. https://search.ebscohost.com/login.aspx?direct=true&db=s3h&AN=152582499&site=ehost-live&scope=site

Felczak, M. (2015). Narratives of Spectatorship: E-sports in Poland NEW PERSPECTIVES IN GAME STUDIES: PROCEEDINGS OF THE CENTRAL AND EASTERN EUROPEAN GAME STUDIES CONFERENCE,

Felczak, M. (2020). Local eSports media analyzed through the circuit of culture framework: Onet-RAS case study. 4th International GamiFIN Conference, GamiFIN 2020,

Fisher, J., & Foust, J. (2022). #GirlGamers, Soldiers, and Public Relations: Analyzing Gender Representation in US Army Esports. JOURNAL OF COMMUNICATION, 72(2), 165-186. https://doi.org/10.1093/joc/jqab051

Freeman, G., & Wohn, D. Y. (2018). Understanding eSports Team Formation and Coordination [Article]. Computer Supported Cooperative Work: CSCW: An International Journal, 27(3-6), 1019-1050. https://doi.org/10.1007/s10606-017-9299-4

Freeman, G., Wohn, D. Y., & Assoc Comp, M. (2017). Social Support in eSports: Building Emotional and Esteem Support from Instrumental Support Interactions in a Highly Competitive Environment CHI PLAY'17: PROCEEDINGS OF THE ANNUAL SYMPOSIUM ON COMPUTER-HUMAN INTERACTION IN PLAY,

Freitas, B. D. A., Contreras-Espinosa, R. S., & Correia, P. A. P. (2022). Esports Sponsorships: The Double-Edged Sword Effect of Having a Very Vocal Audience VIDEOGAME SCIENCES AND ARTS, VJ 2020,

Freitas, B. D. A., Contreras-Espinosa, R. S., & Correia, P. Á. P. (2019). How society’s negative view of videogames can discourage brands from sponsoring esports. In N. Zagalo, L. Costa, A. I. Veloso, & O. Mealha (Eds.), 11th International Conference on Videogame Sciences and Arts, VJ 2019 (Vol. 1164 CCIS, pp. 136-149): Springer Science and Business Media Deutschland GmbH.

Freitas, B. D. A., Contreras-Espinosa, R. S., & Correia, P. Á. P. (2021). A model of the threats that disreputable behavior present to esports sponsors [Article]. Contemporary Management Research, 17(1), 27-64. https://doi.org/10.7903/CMR.20779

Frias, F. J. L. (2022). The “big red bull” in the esports room: Anti-doping, esports, and energy drinks [Article]. Performance Enhancement and Health, 10(1), Article 100205. https://doi.org/10.1016/j.peh.2021.100205

Funk, D. C., Pizzo, A. D., & Baker, B. J. (2021). eSport management: Embracing eSport education and research opportunities. Sport Management Review, 21(1), 7-13. https://doi.org/10.1016/j.smr.2017.07.008

Gainsbury, S. M., Abarbanel, B., & Blaszczynski, A. (2017). GAME ON: COMPARISON OF DEMOGRAPHIC PROFILES, CONSUMPTION BEHAVIORS, AND GAMBLING SITE SELECTION CRITERIA OF ESPORTS AND SPORTS BETTORS. GAMING LAW REVIEW-ECONOMICS REGULATION COMPLIANCE AND POLICY, 21(8), 575-587. https://doi.org/10.1089/glr2.2017.21813

Gainsbury, S. M., Abarbanel, B., & Blaszczynski, A. (2017). INTENSITY AND GAMBLING HARMS: EXPLORING BREADTH OF GAMBLING INVOLVEMENT AMONG ESPORTS BETTORS. GAMING LAW REVIEW-ECONOMICS REGULATION COMPLIANCE AND POLICY, 21(8), 610-615. https://doi.org/10.1089/glr2.2017.21812

García-Lanzo, S., & Chamarro, A. (2019). Basic psychological needs, passion and motivations in amateur and semi-professional eSports players [Article]. Aloma, 37(2), 59-68. https://doi.org/10.51698/aloma.2019.37.2.63-69

Gawrysiak, J., Burton, R., Jenny, S., & Williams, D. (2020). Using Esports Efficiently to Enhance and Extend Brand Perceptions – A Literature Review. Physical Culture & Sport. Studies & Research, 86(1), 1-14. https://search.ebscohost.com/login.aspx?direct=true&db=s3h&AN=143345155&site=ehost-live&scope=site

Gdonteli, K. E. (2020). E-SPORTS IN OLYMPIC GAMES: A GLOBAL TREND AND PROSPECTS. International Sports Law Review Pandektis, 13(1/2), 50-60. https://search.ebscohost.com/login.aspx?direct=true&db=s3h&AN=149237151&site=ehost-live&scope=site

Ghoshal, A. (2019). ETHICS IN ESPORTS. GAMING LAW REVIEW-ECONOMICS REGULATION COMPLIANCE AND POLICY, 23(5), 338-343. https://doi.org/10.1089/glr2.2019.2357

Giakoni-Ramirez, F., Merellano-Navarro, E., & Duclos-Bastias, D. (2022). Professional Esports Players: Motivation and Physical Activity Levels. International Journal of Environmental Research and Public Health, 19(4). https://doi.org/10.3390/ijerph19042256

Goebeler, L., Standaert, W., & Xiao, X. (2021). Hybrid sport configurations: The intertwining of the physical and the digital. 54th Annual Hawaii International Conference on System Sciences, HICSS 2021,

Gong, D. K., Ma, W. Y., Liu, T. J., Yan, Y. N., & Yao, D. Z. (2019). Electronic-Sports Experience Related to Functional Enhancement in Central Executive and Default Mode Areas. Neural Plasticity, 2019. https://doi.org/10.1155/2019/1940123

Greer, N., Rockloff, M., Hing, N., Browne, M., & King, D. L. (2022). Skin Gambling Contributes to Gambling Problems and Harm After Controlling for Other Forms of Traditional Gambling. J Gambl Stud. https://doi.org/10.1007/s10899-022-10111-z

Grushko, A., Morozova, O., Ostapchuk, M., & Korobeynikova, E. (2021). Perceptual-Cognitive Demands of Esports and Team Sports: A Comparative Study. In B. M. Velichkovsky, P. M. Balaban, & V. L. Ushakov (Eds.), 9th International Conference on Cognitive Sciences, Intercognsci 2020 (Vol. 1358 AIST, pp. 36-43): Springer Science and Business Media Deutschland GmbH.

Guo, X. (2021). Research on Advertising Marketing in E-sports Competition in China. 2020 International Conference on New Energy Technology and Industrial Development, NETID 2020,

Gupta, D., Sharma, H., & Gupta, M. (2021). Doping as a barrier in universal acceptance of esports. International Journal of Sports Marketing & Sponsorship. https://doi.org/10.1108/IJSMS-05-2021-0105

Hagiwara, G., Akiyama, D., & Takeshita, S. (2019). Examining effectiveness of e-sports activity in Japan. Journal of Human Sport and Exercise, 14, S1038-S1045. https://doi.org/10.14198/jhse.2019.14.Proc4.66

Hamari, J., & Sjöblom, M. (2017). What is eSports and why do people watch it?. Internet research.

Hamilton, J. (2019). The rise of eSports [Article]. ITNOW, 61(3), 28-29. https://doi.org/10.1093/itnow/bwz068

Hao, L., Lv, Q., Zhang, X., Jiang, Q., Liu, S., & Ping, L. (2020). Conquering Gender Stereotype Threat in "digit Sports": Effects of Gender Swapping on Female Players' Continuous Participation Intention in ESports [Article]. Scientific Programming, 2020, Article 8818588. https://doi.org/10.1155/2020/8818588

Harris, B. C., Hansen, J., Can, O., Rahman, M. W. U., Foxman, M., Cote, A. C., & Fickle, T. (2022). “Starting from scratch to looking really clean and professional”: how students’ productive labor legitimizes collegiate esports [Article]. Critical Studies in Media Communication. https://doi.org/10.1080/15295036.2022.2030484

Himmelstein, D., Liu, Y., & Shapiro, J. L. (2017). An exploration of mental skills among competitive league of legend players [Review]. International Journal of Gaming and Computer-Mediated Simulations, 9(2), 1-21. https://doi.org/10.4018/IJGCMS.2017040101

Hitar-Garcia, J., Moran-Fernandez, L., & Bolon-Canedo, V. (2022). Machine Learning Methods for Predicting League of Legends Game Outcome [Article]. IEEE Transactions on Games. https://doi.org/10.1109/TG.2022.3153086

Hofmann, A. R. (2019). eSport or the disembodiment of sports - A threat to PE classes? International Journal of Physical Education, 56(3), 31-42. https://search.ebscohost.com/login.aspx?direct=true&db=s3h&AN=139605356&site=ehost-live&scope=site

Hollist, K. E. (2015). Time to be grown-ups about video gaming: the rising eSports industry and the need for regulation. Ariz. L. Rev., 57, 823.

Hong, H. J. (2022). eSports: the need for a structured support system for players [Article]. European Sport Management Quarterly. https://doi.org/10.1080/16184742.2022.2028876

Holden, J. T., Edelman, M., & Baker III, T. A. (2020). A short treatise on esports and the law: How America regulates its next national pastime. U. Ill. L. Rev., 509.

Huettermann, M., Trail, G. T., Pizzo, A. D., & Stallone, V. (2020). Esports Sponsorship: An Empirical Examination of Esports Consumers’ Perceptions of Non-Endemic Sponsors [Article]. Journal of Global Sport Management. https://doi.org/10.1080/24704067.2020.1846906

Hughes, D. C., & Orr, W. T. (2019). How Historically Black Colleges and Universities (HBCUs) can benefit from e-sports while adding diversity into the gaming industry. The Sport Journal, 22.

Hutchins, B. (2008). Signs of meta-change in second modernity: the growth of e-sport and the World Cyber Games. NEW MEDIA & SOCIETY, 10(6), 851-869. https://doi.org/10.1177/1461444808096248

Hwang, J. (2022). CHEATING IN E-SPORTS: A PROPOSAL TO REGULATE THE GROWING PROBLEM OF E-DOPING [Article]. Northwestern University Law Review, 116(5), 1283-1318. https://www.scopus.com/inward/record.uri?eid=2-s2.0-85126651136&partnerID=40&md5=23c484c6c073dfd7e1de3dc7faa207db

Igelman, A. A., & Prizant, J. J. (2017). THE CHESS CONUNDRUM: SKILL GAMING AND THE CHALLENGES OF HEAD-TO-HEAD WAGERING. GAMING LAW REVIEW-ECONOMICS REGULATION COMPLIANCE AND POLICY, 21(9), 650-655. https://doi.org/10.1089/glr2.2017.2192

Ikenami, R. K., Lipovaya, V., Da Costa, P. G. F., Silva, É. R., Martins, P. S., Da Silveira Lobo, L., & Duarte, F. J. (2020). A method proposal to support decision-making in unstable ecosystems: Application in the Brazilian eSports ecosystem case [Conference Paper]. International Journal of Technology Management, 82(2), 172-195. https://doi.org/10.1504/IJTM.2020.107857

Iwatsuki, T., Hagiwara, G., & Dugan, M. E. (2022). Effectively optimizing esports performance through movement science principles [Review]. International Journal of Sports Science and Coaching, 17(1), 202-207. https://doi.org/10.1177/17479541211016927

J. S. C, A., M, A., & A. M, G.-T. (2022). Gender influence on brand recommendation at an esports event. Journal of Physical Education & Sport, 22(1), 231-238. https://search.ebscohost.com/login.aspx?direct=true&db=s3h&AN=155075649&site=ehost-live&scope=site

Jang, W., & Byon, K. K. (2019). Antecedents and consequence associated with esports gameplay. International Journal of Sports Marketing and Sponsorship, 21(1), 1-22. https://doi.org/10.1108/ijsms-01-2019-0013

Jang, W. W., & Byon, K. K. (2020). Antecedents of esports gameplay intention: Genre as a moderator [Article]. Computers in Human Behavior, 109, Article 106336. https://doi.org/10.1016/j.chb.2020.106336

Jang, W. W., Byon, K. K., Baker Iii, T. A., & Tsuji, Y. (2021). Mediating effect of esports content live streaming in the relationship between esports recreational gameplay and esports event broadcast [Article]. Sport, Business and Management: An International Journal, 11(1), 89-108. https://doi.org/10.1108/SBM-10-2019-0087

Jenny, S. E., Keiper, M. C., Taylor, B. J., Williams, D. P., Gawrysiak, J., Manning, R. D., & Tutka, P. M. (2018). eSports Venues: A New Sport Business Opportunity. Journal of Applied Sport Management, 10(1), 34-49. https://doi.org/10.18666/jasm-2018-v10-i1-8469

Jenny, S. E., Manning, R. D., Keiper, M. C., & Olrich, T. W. (2016). Virtual(ly) Athletes: Where eSports Fit Within the Definition of “Sport”. Quest, 69(1), 1-18. https://doi.org/10.1080/00336297.2016.1144517

Jeong, I., Nakagawa, K., Osu, R., & Kanosue, K. (2022). Difference in gaze control ability between low and high skill players of a real-time strategy game in esports. PLoS One, 17(3), e0265526. https://doi.org/10.1371/journal.pone.0265526

Jonasson, K., & Thiborg, J. (2010). Electronic sport and its impact on future sport. Sport in Society, 13(2), 287-299. https://doi.org/10.1080/17430430903522996

Jonnalagadda, A., Frosio, I., Schneider, S., McGuire, M., & Kim, J. (2021). Robust Vision-Based Cheat Detection in Competitive Gaming. PROCEEDINGS OF THE ACM ON COMPUTER GRAPHICS AND INTERACTIVE TECHNIQUES, 4(1). https://doi.org/10.1145/3451259

Kane, D., & Spradley, B. D. (2017). Recognizing ESports as a Sport. Sport Journal, 1-1. https://search.ebscohost.com/login.aspx?direct=true&db=s3h&AN=123066341&site=ehost-live&scope=site

Kang, S. K., & Lee, J. H. (2020). An E-sports video highlight generator using win-loss probability model. 35th Annual ACM Symposium on Applied Computing, SAC 2020,

Karhulahti, V.-M. (2017). Reconsidering Esport: Economics and Executive Ownership. Physical Culture & Sport. Studies & Research, 74(1), 43-53. https://search.ebscohost.com/login.aspx?direct=true&db=s3h&AN=124137616&site=ehost-live&scope=site

Katona, A., Spick, R., Hodge, V. J., Demediuk, S., Block, F., Drachen, A., & Walker, J. A. (2019). Time to die: Death prediction in dota 2 using deep learning. 2019 IEEE Conference on Games, CoG 2019,

Ke, X., & Wagner, C. (2020). Global pandemic compels sport to move to esports: understanding from brand extension perspective [Article]. Managing Sport and Leisure, 1-6. https://doi.org/10.1080/23750472.2020.1792801

Ke, X., Wagner, C., & S. Du, H. (2022). Calling for Information Systems Research on Esports: An Overview Study. Communications of the Association for Information Systems, 50, 261-285. https://doi.org/10.17705/1cais.05010

Kee-Young, Y. (2020). POLICIES AND LEGISLATION OF E-SPORTS IN KOREA. International Sports Law Review Pandektis, 13(1/2), 17-29. https://search.ebscohost.com/login.aspx?direct=true&db=s3h&AN=149237148&site=ehost-live&scope=site

Keller, L., Bieleke, M., & Wolff, W. (2021). Bursting balloons - comparison of risk taking between extreme sports, esports, and the general public [Article]. Current Psychology. https://doi.org/10.1007/s12144-021-02616-4

Kelly, S. J., & Van der Leij, D. (2020). A new frontier: alcohol sponsorship activation through esports [Article]. Marketing Intelligence and Planning, 39(4), 533-558. https://doi.org/10.1108/MIP-03-2020-0101

Kempe-Cook, L., Sher, S. T. H., & Su, N. M. (2019). Behind the voices: The practice and challenges of eSports casters. 2019 CHI Conference on Human Factors in Computing Systems, CHI 2019.

Khromov, N., Korotin, A., Lange, A., Stepanov, A., Burnaev, E., & Somov, A. (2019). Esports athletes and players: A comparative study. IEEE Pervasive Computing, 18(3), 31-39.

Kim, J., & Kim, M. (2020). Spectator e-sport and well-being through live streaming services [Article]. Technology in Society, 63, Article 101401. https://doi.org/10.1016/j.techsoc.2020.101401

Kim, Y. (2013). A study of wellness core technology and E-sports. 2013 3rd International Conference on IT Convergence and Security, ICITCS 2013, Macau.

Kim, Y. J., Choi, M. J., & Cha, Y. S. (2012). A study on the impact of e-sports participation by students with mental retardation on their self-confidence and social skills. 4th International Conference on Ubiquitous and Future Networks, ICUFN 2012, Phuket.

Kocak, U. Z. (2021). Are eSports more than just sitting? A study comparing energy expenditure. Journal of Comparative Effectiveness Research, 11(1), 39-45. https://doi.org/10.2217/cer-2021-0223

Kokkinakis, A. V., Demediuk, S., Nölle, I., Olarewaju, O., Patra, S., Robertson, J., York, P., Pedrassoli Chitayat, A. P., Coates, A., Slawson, D., Hughes, P., Hardie, N., Kirman, B., Hook, J., Drachen, A., Ursu, M. F., & Block, F. (2020). DAX: Data-Driven Audience Experiences in Esports. 2020 ACM International Conference on Interactive Media Experiences, IMX 2020,

Komatsu, M., Matsumoto, T., & Prowant, C. (2021). Learning through eSports in innovation practice on electrical technology. 25th KES International Conference on Knowledge-Based and Intelligent Information and Engineering Systems, KES 2021,

Koposov, D., Semenova, M., Somov, A., Lange, A., Stepanov, A., & Burnaev, E. (2020). Analysis of the Reaction Time of eSports Players through the Gaze Tracking and Personality Trait. 29th IEEE International Symposium on Industrial Electronics, ISIE 2020,

Kordyaka, B., Hribersek, S., Kruse, B., & Niehaves, B. (2020). Understanding brand loyalty - The case of the eSports consumer from a relationship quality perspective. 4th International GamiFIN Conference, GamiFIN 2020,

Korotin, A., Khromov, N., Stepanov, A., Lange, A., Burnaev, E., & Somov, A. (2019). Towards understanding of eSports athletes' potentialities: The sensing system for data collection and analysis. 2019 IEEE SmartWorld, Ubiquitous Intelligence and Computing, Advanced and Trusted Computing, Scalable Computing and Communications, Internet of People and Smart City Innovation, SmartWorld/UIC/ATC/SCALCOM/IOP/SCI 2019,

Korotin, A., Stepanov, A., Lange, A., Nikolaev, D., Abramov, S., Klyuchnikov, N., Burnaev, E., & Somov, A. (2021). Assessment of Video Games Players and Teams Behaviour via Sensing and Heterogeneous Data Analysis: Deployment at an eSports Tournament. In S. Paiva, S. I. Lopes, R. Zitouni, N. Gupta, S. F. Lopes, & T. Yonezawa (Eds.), 6th EAI International Conference on Science and Technologies for Smart Cities, SmartCity 2020 (Vol. 372, pp. 409-421): Springer Science and Business Media Deutschland GmbH.

Kosa, G., Feher, G., Horvath, L., Zadori, I., Nemeskeri, Z., Kovacs, M., Fejes, E., Meszaros, J., Banko, Z., & Tibold, A. (2022). Prevalence and Risk Factors of Problematic Internet Use among Hungarian Adult Recreational Esports Players. International Journal of Environmental Research and Public Health, 19(6). https://doi.org/10.3390/ijerph19063204

Kottama, N. R., Sebastian, N., Adrianto, H. A., Harefa, J., & Chowanda, A. (2021). The Mobile Life Simulator for Introducing the Career as a Professional Player in E-Sport. 5th International Conference on Computer Science and Computational Intelligence, ICCSCI 2020,

Kou, Y. (2020). Toxic Behaviors in Team-Based Competitive Gaming: The Case of League of Legends. 7th ACM SIGCHI Annual Symposium on Computer-Human Interaction in Play, CHI PLAY 2020,

Kou, Y., & Gui, X. (2020). Emotion Regulation in eSports Gaming: A Qualitative Study of League of Legends [Article]. Proceedings of the ACM on Human-Computer Interaction, 4(CSCW2), Article 158. https://doi.org/10.1145/3415229

Kozachuk, J., Foroughi, C. K., & Freeman, G. (2016). Exploring electronic sports: An interdisciplinary approach. Human Factors and Ergonomics Society 2016 International Annual Meeting, HFES 2016,

Kozakova, P. (2018). Personal and Psychological Motivators of European Players to Buy Virtual Goods in Free-to-Play Online Games: Czech and Russian Comparison PROCEEDINGS OF THE 4TH INTERNATIONAL CONFERENCE ON EUROPEAN INTEGRATION 2018 (ICEI 2018), PTS 1-3,

Kriglstein, S., Martin-Niedecken, A. L., Turmo Vidal, L., Klarkowski, M., Rogers, K., Turkay, S., Seif El-Nasr, M., Márquez Segura, E., Drachen, A., & Hämäläinen, P. (2021). Special Interest Group: The Present and Future of Esports in HCI. 2021 CHI Conference on Human Factors in Computing Systems: Making Waves, Combining Strengths, CHI EA 2021,

Kunz, R. E., Roth, A., & Santomier, J. P. (2021). A perspective on value co-creation processes in eSports service ecosystems [Article]. Sport, Business and Management: An International Journal. https://doi.org/10.1108/SBM-03-2021-0039

Kwag, S., Lee, W. J., & Ko, Y. D. (2022). Optimal seat allocation strategy for e-sports gaming center [Article]. International Transactions in Operational Research, 29(2), 783-804. https://doi.org/10.1111/itor.12809

Lange, A., Somov, A., Stepanov, A., & Burnaev, E. (2022). Building a Behavioral Profile and Assessing the Skill of Video Game Players. IEEE Sensors Journal, 22(1), 481-488. https://doi.org/10.1109/JSEN.2021.3127083

Lazcano, A., & Avedillo, A. (2021). Equating Esports and Traditional Sports May Facilitate its Regulation Around the World. GAMING LAW REVIEW-ECONOMICS REGULATION COMPLIANCE AND POLICY, 25(10), 459-463. https://doi.org/10.1089/glr2.2021.0016

Lee, S., Bonnar, D., Kim, Y., Lee, Y., Lee, S., Gradisar, M., & Suh, S. (2020). Sleep Characteristics and Risk Factors of Korean Esports Athletes: An Exploratory Study [Article]. Sleep Medicine Research, 11(2), 77-87. https://doi.org/10.17241/SMR.2020.00773

Lee, S., Bonnar, D., Roane, B., Gradisar, M., Jang, E., & Suh, S. (2020). SLEEP CHARACTERISTICS AND MOOD OF PROFESSIONAL ESPORTS ATHLETES: A MULTI-NATIONAL STUDY. SLEEP, 43, A75-A76.

Lee, S. S., Lin, H. Y., Teo, K. H., Tan, W. P., & Lin, C. W. (2018). Discussion on the consumers motivation to watch e-sports game and the follow-up behaviors intention. 2nd International Conference on Education and E-Learning, ICEEL 2018,

Lee, S. W., An, J. W., & Lee, J. Y. (2014, February). The relationship between e-sports viewing motives and satisfaction: The case of League of Legends. In Proceedings of international conference on business, management & corporate social responsibility (pp. 33-36).

Leung, K. M., Wong, M. Y., Ou, K. L., Chung, P. K., & Lau, K. L. (2021). Assessing Esports Participation Intention: The Development and Psychometric Properties of the Theory of Planned Behavior-Based Esports Intention Questionnaire (TPB-Esport-Q). Int J Environ Res Public Health, 18(23). https://doi.org/10.3390/ijerph182312653

Li, L., Uttarapong, J., Freeman, G., & Wohn, D. Y. (2020). Spontaneous, Yet Studious: Esports Commentators' Live Performance and Self-Presentation Practices [Article]. Proceedings of the ACM on Human-Computer Interaction, 4(CSCW2), Article 103. https://doi.org/10.1145/3415174

Lie, H., Lukas, D., Liebig, J., & Nayak, R. (2019). A novel learning-to-rank method for automated camera movement control in e-sports spectating. In G. Warwick, D. Stirling, R. Islam, Y. S. Koh, Z. Islam, C. T. Li, & Y. Zhao (Eds.), 16th Australasian Conference on Data Mining, AusDM 2018 (Vol. 996, pp. 149-160): Springer Verlag.

Lin, Z., & Zhao, Y. (2020). Self-enterprising eSports: Meritocracy, precarity, and disposability of eSports players in China [Article]. International Journal of Cultural Studies, 23(4), 582-599. https://doi.org/10.1177/1367877920903437

Lindsey, M. V. (2016). Gaming before e-sports: Playing with gender in early gaming communities, 1993-2001. In Examining the Evolution of Gaming and Its Impact on Social, Cultural, and Political Perspectives (pp. 170-194). IGI Global. https://doi.org/10.4018/978-1-5225-0261-6.ch008

Lipovaya, V., Costa, P., Grillo, P., Volosiuk, A., & Sopina, A. (2019). eSports: Opportunities for future ergonomic studies. In S. Bagnara, R. Tartaglia, S. Albolino, T. Alexander, & Y. Fujita (Eds.), 20th Congress of the International Ergonomics Association, IEA 2018 (Vol. 824, pp. 1937-1948): Springer Verlag.

Lokhman, N., Karashchuk, O., & Kornilova, O. (2018). Analysis of esports as a commercial activity [Article]. Problems and Perspectives in Management, 16(1), 207-213. https://doi.org/10.21511/ppm.16(1).2018.20

Lu, B., Fa, H. Q., & Yang, Y. (2010). Beijing E-Sports Industry Development Based on SWOT Analysis INTERNATIONAL INNOVATION DESIGN AND MANAGEMENT FORUM AND DESIGN SYMPOSIUM OF CHINESE AROUND THE WORLD IN 2010,

Lu, Z. (2016). From E-Heroin to E-Sports: The Development of Competitive Gaming in China. International Journal of the History of Sport, 33(18), 2186-2206. https://search.ebscohost.com/login.aspx?direct=true&db=s3h&AN=125437857&site=ehost-live&scope=site

Lukowicz, K., & Strzelecki, A. (2020). USER SATISFACTION ON SOCIAL MEDIA PROFILE OF E-SPORTS ORGANIZATION. MARKETING AND MANAGEMENT OF INNOVATIONS(4), 61-75. https://doi.org/10.21272/mmi.2020.4-05

Macey, J., Tyrvainen, V., Pirkkalainen, H., & Hamari, J. (2020). Does esports spectating influence game consumption? BEHAVIOUR & INFORMATION TECHNOLOGY. https://doi.org/10.1080/0144929X.2020.1797876

Maciej, B., Kosakowski, M., & Kaczmarek, L. D. (2020). Social challenge and threat predict performance and cardiovascular responses during competitive video gaming. Psychology of Sport and Exercise, 46. https://doi.org/10.1016/j.psychsport.2019.101584

Marcin, W., Michał, D. K., Małgorzata, Ć., & Przemysław, C. (2019). Analysis of matchmaking optimization systems potential in mobile eSports. 52nd Annual Hawaii International Conference on System Sciences, HICSS 2019,

Marta, R. F., Fernando, J., Sampurna, A., Jarata, J. R. B., & Syarnubi, K. L. (2021). Interactivity in E-Sport Future Learning from the Choices and Attributes Perspectives of Online News [Article]. Review of International Geographical Education Online, 11(4), 1192-1202. https://doi.org/10.33403/rigeo.8006835

Martinelli, D. (2017). SKIN GAMBLING: HAVE WE FOUND THE MILLENNIAL GOLDMINE OR IMMINENT TROUBLE? GAMING LAW REVIEW-ECONOMICS REGULATION COMPLIANCE AND POLICY, 21(8), 557-565. https://doi.org/10.1089/glr2.2017.21814

Mateo-Orcajada, A., Abenza-Cano, L., & Vaquero-Cristóbal, R. (2022). Analyzing the changes in the psychological profile of professional League of Legends players during competition [Article]. Computers in Human Behavior, 126, Article 107030. https://doi.org/10.1016/j.chb.2021.107030

Mathonat, R., Boulicaut, J. F., & Kaytoue, M. (2020). A Behavioral Pattern Mining Approach to Model Player Skills in Rocket League. 2020 IEEE Conference on Games, CoG 2020,

McCauley, B., Nguyen, T. H., McDonald, M., & Wearing, S. (2020). Digital gaming culture in Vietnam: an exploratory study. Leisure Studies, 39(3), 372-386. https://doi.org/10.1080/02614367.2020.1731842

McCutcheon, C., Hitchens, M., & Drachen, A. (2018). eSport vs irlSport. In M. Inami, T. Romao, A. D. Cheok, & A. D. Cheok (Eds.), 14th International Conference on Advances in Computer Entertainment Technology, ACE 2017 (Vol. 10714 LNCS, pp. 531-542): Springer Verlag.

Melentev, N., Somov, A., Burnaev, E., Strelnikova, I., Strelnikova, G., Melenteva, E., & Menshchikov, A. (2020). ESports Players Professional Level and Tiredness Prediction using EEG and Machine Learning. 2020 IEEE Sensors, SENSORS 2020,

Milić, M. K. (2020). E-sport during the covid 19 pandemic and its negative impact on child health [Article]. Sportske Nauke i Zdravlje, 10(2), 193-199. https://doi.org/10.7251/SSH2002193M

Mizumatsu, Y., Oda, R., & Kajinami, T. (2018). A User Interface to Visualize the Viewing Tendencies of e-Sports Players. 7th IEEE Global Conference on Consumer Electronics, GCCE 2018,

Moen, F., Vatn, M., Olsen, M., Haugan, J. A., & Skalicka, V. (2022). Sleep Characteristics in Esport Players and Associations With Game Performance: Residual Dynamic Structural Equation Modeling [Article]. FRONTIERS IN SPORTS AND ACTIVE LIVING, 3, Article 697535. https://doi.org/10.3389/fspor.2021.697535

Murakami, K., & Miyachi, H. (2022). A Study on the Impact of High Refresh-Rate Displays on Scores of eSports. In L. Barolli, H. Chen, & H. Miwa (Eds.), 24th International Conference on Network-Based Information Systems, NBiS-2021 (Vol. 313, pp. 283-288): Springer Science and Business Media Deutschland GmbH.

Murakami, K., Miyashita, K., & Miyachi, H. (2021). A Study on the Relationship Between Refresh-Rate of Display and Reaction Time of eSports. In L. Barolli, M. Ikeda, M. Takizawa, T. Yoshihisa, & F. Amato (Eds.), 15th International Conference on P2P, Parallel, Grid, Cloud and Internet Computing, 3PGCIC 2020, held in conjunction with the 15th International Conference on Broadband and Wireless Computing, Communication and Applications, BWCCA 2020 (Vol. 158 LNNS, pp. 339-347): Springer Science and Business Media Deutschland GmbH.

Murray, S., Birt, J., & Blakemore, S. (2020). eSports diplomacy: towards a sustainable ‘gold rush’ [Article]. Sport in Society. https://doi.org/10.1080/17430437.2020.1826437

N. S, K. (2020). ESPORTS AND ITS REINFORCEMENT OF GENDER DIVIDES. Marquette Sports Law Review, 30(2), 347-369. https://search.ebscohost.com/login.aspx?direct=true&db=s3h&AN=146163859&site=ehost-live&scope=site

Nagorsky, E., & Wiemeyer, J. (2021). The structure of performance and training in esports (vol 15, e0237584, 2020). PLoS One, 16(4). https://doi.org/10.1371/journal.pone.0250316

Naweed, A., Irwin, S. V., & Lastella, M. (2020). Varieties of (Un)sportsmanlike Conduct in the FPS Esports Genre: A Taxonomic Classification of ‘Esportsmanship’ [Article]. Journal of Global Sport Management. https://doi.org/10.1080/24704067.2020.1846907

Neus, F., Nimmermann, F., Wagner, K., & Schramm-Klein, H. (2019). Differences and similarities in motivation for offline and online eSports event consumption. 52nd Annual Hawaii International Conference on System Sciences, HICSS 2019,

Novak, A. R., Bennett, K. J., Pluss, M. A., & Fransen, J. (2020). Performance analysis in esports: modelling performance at the 2018 League of Legends World Championship. International Journal of Sports Science & Coaching, 15(5-6), 809-817. https://doi.org/10.1177/1747954120932853

Oleksandr, P., Natalia, S., & Anastasia, V. (2022). Less than Attractiveness of e-Sports Games for Female Audience-Journalism. INTERNATIONAL JOURNAL OF COMPUTER SCIENCE AND NETWORK SECURITY, 22(2), 57-62. https://doi.org/10.22937/IJCSNS.2022.22.2.8

Ostergaard, S. N., & Ronde, M. (2021). Skin Betting Within Esports in Denmark. GAMING LAW REVIEW-ECONOMICS REGULATION COMPLIANCE AND POLICY, 25(10), 480-484. https://doi.org/10.1089/glr2.2021.0011

Palanichamy, T., Sharma, M. K., Sahu, M., & Kanchana, D. M. (2020). Influence of Esports on stress: A systematic review. Ind Psychiatry J, 29(2), 191-199. https://doi.org/10.4103/ipj.ipj_195_20

Palma-Ruiz, J. M., Torres-Toukoumidis, A., Gonzalez-Moreno, S. E., & Valles-Baca, H. G. (2022). An overview of the gaming industry across nations: using analytics with power BI to forecast and identify key influencers. Heliyon, 8(2). https://doi.org/10.1016/j.heliyon.2022.e08959

Pan, Z., He, G., Su, S., Li, X., & Pan, J. (2006). Virtual network marathon: Fitness-oriented E-sports in distributed virtual environment (A. International Journal of, Computer, R. International Journal of Virtual, C. Nature Science Foundation of, C. Peking University, C. Tsinghua University, & C. Zhegeng University, Trans.). In 12th International Conference on Virtual Systems and Multimedia, VSMM 2006 (Vol. 4270 LNCS, pp. 520-529). Xi'an: Springer Verlag.

Paravizo, E., & de Souza, R. L. R. (2021). Towards Improving Esports’ Working Conditions: Insights on Role of a Professional Players’ Association. In N. L. Black, W. P. Neumann, & I. Noy (Eds.), 21st Congress of the International Ergonomics Association, IEA 2021 (Vol. 219 LNNS, pp. 755-761): Springer Science and Business Media Deutschland GmbH.

Paravizo, E., & de Souza, R. R. L. (2019). Playing for real: An exploratory analysis of professional esports athletes’ work. In Y. Fujita, S. Bagnara, R. Tartaglia, S. Albolino, & T. Alexander (Eds.), 20th Congress of the International Ergonomics Association, IEA 2018 (Vol. 822, pp. 507-515): Springer Verlag.

Parshakov, P., Paklina, S., Coates, D., & Chadov, A. (2020). Does video games’ popularity affect unemployment rate? Evidence from macro-level analysis [Article]. Journal of Economic Studies, 48(4), 817-835. https://doi.org/10.1108/JES-07-2019-0339

Pedraza-Ramirez, I., Musculus, L., Raab, M., & Laborde, S. (2020). Setting the scientific stage for esports psychology: A systematic review. International Review of Sport and Exercise Psychology, 13(1), 319-352.

Pizzo, A., Baker, B., Na, S., Lee, M., Kim, D., & Funk, D. (2018). eSport vs sport: a comparison of spectator motives. Faculty/Researcher Works.

Pizzo, A. D., Kunkel, T., Jones, G. J., Baker, B. J., & Funk, D. C. (2022). The strategic advantage of mature-stage firms: Digitalization and the diversification of professional sport into esports [Article]. Journal of Business Research, 139, 257-266. https://doi.org/10.1016/j.jbusres.2021.09.057

Pizzo, A. D., Sangwon, N., Baker, B. J., Mi Ae, L., Doohan, K., & Funk, D. C. (2018). eSport vs. Sport: A Comparison of Spectator Motives. Sport Marketing Quarterly, 27(2), 108-123. https://search.ebscohost.com/login.aspx?direct=true&db=s3h&AN=130519559&site=ehost-live&scope=site

Pluss, M. A., Bennett, K. J. M., Novak, A. R., Panchuk, D., Coutts, A. J., & Fransen, J. (2019). Esports: The Chess of the 21st Century. Front Psychol, 10, 156. https://doi.org/10.3389/fpsyg.2019.00156

Pluss, M. A., Novak, A. R., Bennett, K. J. M., Panchuk, D., Coutts, A. J., & Fransen, J. (2022). The reliability and validity of mobalytics proving ground as a perceptual-motor skill assessment for esports [Article]. International Journal of Sports Science and Coaching. https://doi.org/10.1177/17479541221086793

Polman, R., Trotter, M., Poulus, D., & Borkoles, E. (2018). eSport: Friend or Foe? In M. Oliveira, J. Baalsrud Hauge, S. Gobel, A. Garcia-Agundez, T. Tregel, P. Caserman, T. Marsh, & M. Ma (Eds.), 4th International Joint Conference on Serious Games, JCSG 2018 (Vol. 11243 LNCS, pp. 3-8): Springer Verlag.

Poulus, D. R., Coulter, T. J., Trotter, M. G., & Polman, R. (2021). A qualitative analysis of the perceived determinants of success in elite esports athletes. Journal of Sports Sciences. https://doi.org/10.1080/02640414.2021.2015916

Poulus, D. R., Coulter, T. J., Trotter, M. G., & Polman, R. (2022). Longitudinal analysis of stressors, stress, coping and coping effectiveness in elite esports athletes [Article]. Psychology of Sport and Exercise, 60, Article 102093. https://doi.org/10.1016/j.psychsport.2021.102093

Priladha, A., & Setiyadi, A. (2019). Designing Information System Recruitment Professional Gamers Web-Based. 2nd International Conference on Informatics, Engineering, Science, and Technology, INCITEST 2019,

Pu, H., Kim, J., & Daprano, C. (2021). Can esports substitute traditional sports? The convergence of sports and video gaming during the pandemic and beyond [Article]. Societies, 11(4), Article 129. https://doi.org/10.3390/soc11040129

Qian, T. Y., Matz, R., Luo, L., & Xu, C. (2022). Gamification for value creation and viewer engagement in gamified livestreaming services: The moderating role of gender in esports [Article]. Journal of Business Research, 145, 482-494. https://doi.org/10.1016/j.jbusres.2022.02.082

Qian, X., Sifa, R., Liu, X., Ganguly, S., Yadamsuren, B., Klabjan, D., Drachen, A., & Demediuk, S. (2022). Anomaly Detection in Player Performances in Multiplayer Online Battle Arena Games.

Railsback, D., & Caporusso, N. (2019). Investigating the human factors in esports performance. In T. Z. Ahram (Ed.), AHFE 2018 International Conferences on Human Factors and Wearable Technologies, and Human Factors in Game Design and Virtual Environments, 2018 (Vol. 795, pp. 325-334): Springer Verlag.

Raisinghani, M. S. (2020). The expert opinion report with Mr. Clarence James (CJ) Collins, Managing Partner at CLT E-Sports & Charlotte Phoenix, CLT, USA [Note]. Journal of Information Technology Case and Application Research, 22(1), 67-69. https://doi.org/10.1080/15228053.2020.1742450

Rambusch, J., Jakobsson, P., & Pargman, D. (2007). Exploring E-sports: A case study of gameplay in Counter-strike. 3rd Digital Games Research Association International Conference: ""Situated Play"", DiGRA 2007, Tokyo.

Riatti, P., & Thiel, A. (2021). The societal impact of electronic sport: a scoping review [Review]. German Journal of Exercise and Sport Research. https://doi.org/10.1007/s12662-021-00784-w

Richard, J., Ivoska, W., & Derevensky, J. (2021). TOWARDS AN UNDERSTANDING OF ESPORTS GAMBLING: DEMOGRAPHIC AND CLINICAL CHARACTERISTICS OF YOUTH ESPORTS BETTORS. GAMING LAW REVIEW-ECONOMICS REGULATION COMPLIANCE AND POLICY, 25(10), 426-434. https://doi.org/10.1089/glr2.2021.0013

Ridenhour, K. F. (2020). Traditional sports and esports: The path to collective bargaining [Review]. Iowa Law Review, 105(4), 1857-1897. https://www.scopus.com/inward/record.uri?eid=2-s2.0-85090844886&partnerID=40&md5=317997f2d1c98f7c116cb1094f456353

Rizzi, A., Serao, N., & Nowak, L. (2019). Esports in Italy: An industry ready to take off (or still in search of its regulatory soul)? [Article]. Interactive Entertainment Law Review, 2(1), 49-42. https://doi.org/10.4337/ielr.2019.01.04

Robinson, R., Isbister, K., & Hammer, J. (2019). All the World (Wide Web)'s a stage: A workshop on live streaming. 2019 CHI Conference on Human Factors in Computing Systems, CHI EA 2019,

Rodrigues, E., & Filgueiras, E. (2020). eSports: How Do Video Game Aspects Define Competitive Gaming Streams and Spectatorship. In A. Marcus & E. Rosenzweig (Eds.), 9th International Conference on Design, User Experience, and Usability, DUXU 2020, held as part of the 22nd International Conference on Human-Computer Interaction, HCII 2020 (Vol. 12201 LNCS, pp. 506-516): Springer.

Rogers, R., Farquhar, L., & Mummert, J. (2020). Audience response to endemic and non-endemic sponsors of esports events. International Journal of Sports Marketing and Sponsorship, 21(3), 561-576. https://doi.org/10.1108/ijsms-09-2019-0107

Rogers, R., Farquhar, L., & Mummert, J. (2020). Motivational Differences Among Viewers of Traditional Sports, Esports, and NBA 2K League [Article]. Communication and Sport. https://doi.org/10.1177/2167479520942738

Rogstad, E. T. (2021). Gender in eSports research: a literature review [Article]. European Journal for Sport and Society. https://doi.org/10.1080/16138171.2021.1930941

Rojas-Valverde, D., Fallas-Campos, A., & Alpizar-Alpizar, M. (2020). ESPORTS IN TIMES OF A GLOBAL PANDEMIC: OPPORTUNITIES AND FUTURE CHALLENGES WHEN TRANSFORMING GAMING INTO A SPORT IN COSTA RICA. PENSAR EN MOVIMIENTO-REVISTA DE CIENCIAS DEL EJERCICIO Y LA SALUD, 18(2). https://doi.org/10.15517/pensarmov.v18i2.43332

Rossi, R., Nairn, A., Smith, J., & Inskip, C. (2021). “Get a £10 Free Bet Every Week!”—Gambling Advertising on Twitter: Volume, Content, Followers, Engagement, and Regulatory Compliance [Article]. Journal of Public Policy and Marketing, 40(4), 487-504. https://doi.org/10.1177/0743915621999674

Rudolf, K., Soffner, M., Bickmann, P., Froböse, I., Tholl, C., Wechsler, K., & Grieben, C. (2022). Media Consumption, Stress and Wellbeing of Video Game and eSports Players in Germany: The eSports Study 2020. Front Sports Act Living, 4, 665604. https://doi.org/10.3389/fspor.2022.665604

Ruvalcaba, O., Shulze, J., Kim, A., Berzenski, S. R., & Otten, M. P. (2018). Women’s Experiences in eSports: Gendered Differences in Peer and Spectator Feedback During Competitive Video Game Play. Journal of Sport and Social Issues, 42(4), 295-311. https://doi.org/10.1177/0193723518773287

Ryzhov, I. O., Tariq, A., & Powell, W. B. (2011). May the best man win: Simulation optimization for match-making in e-sports. 2011 Winter Simulation Conference, WSC 2011, Phoenix, AZ.

Saarikoski, P., Suominen, J., & Reunanen, M. (2017). The gamification of digital gaming-video game competitions and high score tables as a prehistory of e-sports in Finland in the 1980s and early 1990s. 1st International GamiFIN Conference, GamiFIN 2017,

Sabtan, B., Cao, S., & Paul, N. (2022). Current practice and challenges in coaching Esports players: An interview study with league of legends professional team coaches [Article]. Entertainment Computing, 42, Article 100481. https://doi.org/10.1016/j.entcom.2022.100481

Sainz, I., Collado-Mateo, D., & Del Coso, J. (2020). Effect of acute caffeine intake on hit accuracy and reaction time in professional e-sports players. PHYSIOLOGY & BEHAVIOR, 224. https://doi.org/10.1016/j.physbeh.2020.113031

Savas, D., Murat, S. H., Cilem, B., & Gunseli, D. (2020). E-Sports Education and Development in a Global World. AMBIENT SCIENCE, 7, 237-242. https://doi.org/10.21276/ambi.2020.07.sp1.ga03

Schaeperkoetter, C. C., Oja, B., Mays, J., Krueger, K., Hyland, S. T., Christian, R., Wilkerson, Z., & Bass, J. R. (2017). The "New" Student-Athlete: An Exploratory Examination of Scholarship eSports Players. Journal of Intercollegiate Sport, 10(1), 1-21. https://search.ebscohost.com/login.aspx?direct=true&db=s3h&AN=124181296&site=ehost-live&scope=site

Scholz, T. M. (2012, December). New broadcasting ways in IPTV–The case of the Starcraft broadcasting scene. In World media economics & management conference.

Scholz, T. M. (2019). eSports is business: Management in the world of competitive gaming [Book]. Springer International Publishing. https://doi.org/10.1007/978-3-030-11199-1

Scholz, T., Völkel, L., & Uebach, C. (2021). Sportification of esports-a systematization of sport-teams entering the esports ecosystem. International Journal of Esports, 2(2).

Schubert, M., Drachen, A., & Mahlmann, T. (2016). Esports analytics through encounter detection. In MIT Sloan Sports Analytics Conference. MIT Sloan.

Schwartz, D. G. (2017). HISTORICAL PARALLELS BETWEEN TOURNAMENT POKER AND ESPORTS. GAMING LAW REVIEW-ECONOMICS REGULATION COMPLIANCE AND POLICY, 21(10), 730-744. https://doi.org/10.1089/glr2.2017.21103

Schwartz, D. G. (2017). RESEARCH (IN)COMPLETE: AN EXPLORATORY HISTORY OF COMPETITIVE VIDEO GAMING. GAMING LAW REVIEW-ECONOMICS REGULATION COMPLIANCE AND POLICY, 21(8), 542-556. https://doi.org/10.1089/glr2.2017.2185

Scott, M. J., Summerley, R., Besombes, N., Connolly, C., Gawrysiak, J., Halevi, T., Jenny, S. E., Miljanovic, M., Stange, M., Taipalus, T., & Williams, J. P. (2021). Foundations for Esports Curricula in Higher Education. 2021 Working Group Reports on Innovation and Technology in Computer Science Education, ITiCSE-WGR 2021,

Semenova, M., Lange, A., Koposov, D., Somov, A., & Burnaev, E. (2020). Personality Traits and Coping Strategies of eSports Players. 7th IEEE International Conference on Behavioural and Social Computing, BESC 2020,

Seo, Y. (2013). Electronic sports: A new marketing landscape of the experience economy. Journal of Marketing Management, 29(13-14), 1542-1560. https://doi.org/10.1080/0267257x.2013.822906

Seo, Y. (2016). Professionalized consumption and identity transformations in the field of eSports. Journal of Business Research, 69(1), 264-272. https://doi.org/10.1016/j.jbusres.2015.07.039

Seo, Y., & Jung, S.-U. (2016). Beyond solitary play in computer games: The social practices of eSports. Journal of Consumer Culture, 16(3), 635-655. https://doi.org/10.1177/1469540514553711

Shum, H. L., Lee, C. H., & Cheung, J. C. S. (2021). Should Esports Be a Co-Curricular Activity in School? CHILDREN & SCHOOLS, 43(1), 61-63. https://doi.org/10.1093/cs/cdaa028

Silva, V. F., Silva, É. M., Lima, K. P., & Scalon, J. D. (2021). Performance of jungler’s champions in the game legue of legends® [Article]. Revista Brasileira de Biometria, 39(1), 114-121. https://doi.org/10.28951/RBB.V39I1.508

Skubida, D. (2016). Can Some Computer Games Be a Sport? International Journal of Gaming and Computer-Mediated Simulations, 8(4), 38-52. https://doi.org/10.4018/ijgcms.2016100103

Soler-Dominguez, J. L., & Gonzalez, C. (2021). Using EEG and gamified neurofeedback environments to improve eSports performance: Project neuroprotrainer. 16th International Joint Conference on Computer Vision, Imaging and Computer Graphics Theory and Applications, VISIGRAPP 2021,

Standaert, W., & Jarvenpaa, S. L. (2016). Formula E: Next generation motorsport with next generation Fans123. 2016 International Conference on Information Systems, ICIS 2016,

Stepanov, A., Lange, A., Khromov, N., Korotin, A., Burnaev, E., & Somov, A. (2019). Sensors and game synchronization for data analysis in esports. 17th IEEE International Conference on Industrial Informatics, INDIN 2019,

Stoever, J. K. (2021). Title IX, Esports, and #EToo. GEORGE WASHINGTON LAW REVIEW, 89(4), 857-931.

Suh, M., Ahn, J., Kim, E., & Um, S. (2008). A Study on the Various Attributes of E-Sport Influencing Flow and Identification. JOURNAL OF GLOBAL SCHOLARS OF MARKETING SCIENCE, 18(1), 59-80. https://doi.org/10.1080/12297119.2008.9707277

Sylvester, R., & Rennie, P. (2017). THE WORLD'S FASTEST-GROWING SPORT: MAXIMIZING THE ECONOMIC SUCCESS OF ESPORTS WHILST BALANCING REGULATORY CONCERNS AND ENSURING THE PROTECTION OF THOSE INVOLVED. GAMING LAW REVIEW-ECONOMICS REGULATION COMPLIANCE AND POLICY, 21(8), 625-629. https://doi.org/10.1089/glr2.2017.21811

Szablewicz, M. (2011). From addicts to athletes: Participation in the discursive construction of digital games in urban China. AoIR Selected Papers of Internet Research.

Szot, M., Karpecka-Galka, E., Drozdz, R., & Fraczek, B. (2022). Can Nutrients and Dietary Supplements Potentially Improve Cognitive Performance Also in Esports? HEALTHCARE, 10(2). https://doi.org/10.3390/healthcare10020186

Taylor, N. T. (2011). Play globally, act locally: The standardization of pro Halo 3 gaming. International Journal of Gender, Science and Technology, 3(1).

Taylor, N. (2015). Play to the camera. Convergence: The International Journal of Research into New Media Technologies, 22(2), 115-130. https://doi.org/10.1177/1354856515580282

Taylor, N. T. (2016). Now you’re playing with audience power: the work of watching games. Critical Studies in Media Communication, 33(4), 293-307. https://doi.org/10.1080/15295036.2016.1215481

Taylor, N., Szablewicz, M., Bowman, N., & Harper, T. (2013). Watching the watchers: New perspectives on spectatorship, gaming and online media. AoIR Selected Papers of Internet Research.

Taylor, T. L. (2020). The Rise of Massive Multiplayer Online Games, Esports, and Game Live Streaming An Interview with T. L.Taylor. AMERICAN JOURNAL OF PLAY, 12(2), 107-116.

Taylor, T. L., & Taylor, T. L. (2012). Raising the Stakes: E-Sports and the Professionalization of Computer Gaming.

The Mission Value of Collegiate Esports. (2021). Sport Journal, 21, N.PAG-N.PAG. https://search.ebscohost.com/login.aspx?direct=true&db=s3h&AN=149507469&site=ehost-live&scope=site

Toomey, R. P. (2019). UPHOLDING THE INTEGRITY OF ESPORTS TO SUCCESSFULLY AND SAFELY LEGITIMIZE ESPORTS WAGERING. GAMING LAW REVIEW-ECONOMICS REGULATION COMPLIANCE AND POLICY, 23(1), 12-18. https://doi.org/10.1089/glr2.2019.23112

Törhönen, M., Sjöblom, M., Vahlo, J., & Hamari, J. (2020). View, play and pay? - The relationship between consumption of gaming video content and video game playing and buying. 53rd Annual Hawaii International Conference on System Sciences, HICSS 2020,

Tregel, T., Sarpe-Tudoran, T., Müller, P. N., & Göbel, S. (2021). Analyzing Game-Based Training Methods for Selected Esports Titles in Competitive Gaming. In B. Fletcher, M. Ma, S. Gobel, J. Baalsrud Hauge, & T. Marsh (Eds.), 7th Joint International Conference on Serious Games, JCSG 2021 (Vol. 12945 LNCS, pp. 213-228): Springer Science and Business Media Deutschland GmbH.

Trotter, M., Davis, P., Coutler, T., Poulus, D., & Polman, R. (2021). Social support, self-regulation, and psychological skill use in e-athletes. International Journal of Sport and Exercise Psychology, 19, S63-S64.

Trotter, M. G., Coulter, T. J., Davis, P. A., Poulus, D. R., & Polman, R. (2020). The Association between Esports Participation, Health and Physical Activity Behaviour. Int J Environ Res Public Health, 17(19). https://doi.org/10.3390/ijerph17197329

Türkay, S., Formosa, J., Adinolf, S., Cuthbert, R., & Altizer, R. (2020). See No Evil, Hear No Evil, Speak No Evil: How Collegiate Players Define, Experience and Cope with Toxicity. 2020 ACM CHI Conference on Human Factors in Computing Systems, CHI 2020,

ValladÃo, S. P., Middleton, J., & Andre, T. L. (2020). Esport: Fortnite Acutely Increases Heart Rate of Young Men. Int J Exerc Sci, 13(6), 1217-1227.

Van Damme, S., Torres Vega, M., Heyse, J., De Backere, F., & De Turck, F. (2020). A low-complexity psychometric curve-fitting approach for the objective quality assessment of streamed game videos [Article]. Signal Processing: Image Communication, 88, Article 115954. https://doi.org/10.1016/j.image.2020.115954

Vansyngel, S., Velpry, A., & Besombes, N. (2018). French esports institutionalization. 2nd International GamiFIN Conference, GamiFIN 2018,

Vera, J. A. C., Terron, J. M. A., & Garcia, S. G. (2018). Following the Trail of eSports: The Multidisciplinary Boom of Research on the Competitive Practice of Video Games. International Journal of Gaming and Computer-Mediated Simulations, 10(4), 42-61. https://doi.org/10.4018/IJGCMS.2018100103

Vieira, J., & Luwes, N. (2020). An Image processing Player Acquisition and Tracking System for E-sports. 5th International Conference on Cloud Computing and Artificial Intelligence: Technologies and Applications, CloudTech 2020.

Wagner, M. G. (2006, June). On the Scientific Relevance of eSports. In International conference on internet computing (pp. 437-442).

Walton, D. R., Lower-Hoppe, L. M., & Horger, M. (2020). Do Esports Classify as Intercollegiate Sport? Legal Analysis of Title IX. Journal of Issues in Intercollegiate Athletics, 94-118. https://search.ebscohost.com/login.aspx?direct=true&db=s3h&AN=143750049&site=ehost-live&scope=site

Wang, H., Huo, H., & Zhang, D. (2021). Structural analysis of e-sports industrial association network based on complex network theory [Article]. Revista de Psicologia del Deporte, 30(2), 298-308. https://www.scopus.com/inward/record.uri?eid=2-s2.0-85115303788&partnerID=40&md5=7b7bb7025b96c8a8a42eb6ab952cf1ce

Wanyi, T. (2018). Understanding Esports from the Perspective of Team Dynamics. Sport Journal, 1-1. https://search.ebscohost.com/login.aspx?direct=true&db=s3h&AN=131066369&site=ehost-live&scope=site

Watson, B., Spjut, J., Kim, J., Listman, J., Kim, S., Wimmer, R., Putrino, D., & Lee, B. (2021). Esports and High Performance HCI. 2021 CHI Conference on Human Factors in Computing Systems: Making Waves, Combining Strengths, CHI EA 2021.

Weiss, T. (2008). Cultural influences on hedonic adoption behavior: Propositions regarding the adoption of competitive video and computer online gaming.

Weiss, T. (2011). Fulfilling the needs of esports consumers: A uses and gratifications perspective. 24th Bled eConference - eFuture: Creating Solutions for the Individual, Organisations and Society,

Werder, K. (2022). Esport [Note]. Business and Information Systems Engineering. https://doi.org/10.1007/s12599-022-00748-w

Whalen, S. J. (2013). Cyberathletes’ lived experience of video game tournaments. PhD diss., University of Tennessee.

Witkowski, E. (2012). On the Digital Playing Field. Games and Culture, 7(5), 349-374. https://doi.org/10.1177/1555412012454222

Wohn, D. Y., & Freeman, G. (2019). Live Streaming, Playing, and Money Spending Behaviors in eSports. Games and Culture, 15(1), 73-88. https://doi.org/10.1177/1555412019859184

Wong, M. Y. C., Chung, P. K., Ou, K. L., & Leung, K. M. (2021). Perception of Hong Kong Teenagers and Young Adults on Esports Participation: A Qualitative Study Using Theory of Planned Behavior. Frontiers in Psychology, 12. https://doi.org/10.3389/fpsyg.2021.650000

Woonhyuk, B., Park, A., Jongin, K., & Keechul, J. (2007). e-Sports live: e-Sports relay broadcasting on demand. In Symposium on Human Interface 2007 (Vol. 4558 LNCS, pp. 685-693). Beijing.

Wooyoung, J., & Byon, K. K. (2021). Investigation of eSports Playing Intention Formation: The Moderating Impact of Gender. Sport Marketing Quarterly, 30(3), 193-206. https://search.ebscohost.com/login.aspx?direct=true&db=s3h&AN=152483515&site=ehost-live&scope=site

Wu, M., Lee, J. S., & Steinkuehler, C. (2021). Understanding tilt in esports: A study on young league of legends players. 2021 CHI Conference on Human Factors in Computing Systems: Making Waves, Combining Strengths, CHI 2021,

Xenopoulos, P., Doraiswamy, H., & Silva, C. (2020). Valuing Player Actions in Counter-Strike: Global Offensive. 8th IEEE International Conference on Big Data, Big Data 2020,

Xiaoxian, L. (2020). E-Sports Hotel, a Brand New Personalized Hotel in the Age of E-Sports. In E. Markopoulos, R. S. Goonetilleke, A. G. Ho, & Y. Luximon (Eds.), AHFE Virtual Conference on Creativity, Innovation and Entrepreneurship and the Virtual Conference on Human Factors in Communication of Design, 2020 (Vol. 1218 AISC, pp. 213-218): Springer.

Xiuqi, Z., Do Young, P., & Manoli, A. E. (2021). Developing a Conceptual Model of Service Quality for eSports. Quest (00336297), 73(4), 375-390. https://search.ebscohost.com/login.aspx?direct=true&db=s3h&AN=154721576&site=ehost-live&scope=site

Yamanaka, G. K., Campos, M. V. S., Roble, O. J., & Mazzei, L. C. (2021). eSport: a state-of-the-art review based on bibliometric analysis. Journal of Physical Education & Sport, 21(6), 3547-3555. https://search.ebscohost.com/login.aspx?direct=true&db=s3h&AN=154466456&site=ehost-live&scope=site

Yan, J. (2018). How Does Match-Fixing Inform Computer Game Security? In V. Matyas, P. Svenda, J. Anderson, B. Christianson, & F. Stajano (Eds.), 26th International Workshop on Security Protocols, 2018 (Vol. 11286 LNCS, pp. 166-170): Springer Verlag.

Yan, S. A., & Mawhorter, P. (2020). Twitter sentiment analysis: Fan engagement in esportsmatches. 13th IADIS International Conference ICT, Society and Human Beings 2020, ICT 2020 and 6th IADIS International Conference Connected Smart Cities 2020, CSC 2020 and 17th IADIS International Conference Web Based Communities and Social Media 2020, WBC 2020, Part of the 14th Multi Conference on Computer Science and Information Systems, MCCSIS 2020,

Yang, A., Liu, D., & Santhanam, R. (2021). The Impact of Role Coordination on Virtual Team Performance and Player Retention in Esports. In A. Garimella, P. Karhade, A. Kathuria, X. Liu, J. Xu, & K. Zhao (Eds.), 19th Workshop on e-Business, WeB 2020 (Vol. 418, pp. 121-128): Springer Science and Business Media Deutschland GmbH.

Yin, K. Y., Zi, Y. H., Zhuang, W., Gao, Y., Tong, Y., Song, L. J., & Liu, Y. (2020). Linking Esports to health risks and benefits: Current knowledge and future research needs. Journal of Sport and Health Science, 9(6), 485-488. https://doi.org/10.1016/j.jshs.2020.04.006

Zang, L. H., Wu, J., & Li, Y. B. (2007). Research on current situation of E-sports in Urumqi, Xinjiang PROCEEDINGS OF UK-CHINA SPORTS ENGINEERING WORKSHOP,

Zhang, G. (2012). Bibliometric analysis on E-sports in China (S. Huazhong University of, Technology, & A. International Communication Sciences, Trans.). In 2012 2nd International Conference on Advances in Computer Science and Engineering, CES 2012 (Vol. 141 AISC, pp. 111-118). Sanya.

Zhao, Y., & Lin, Z. (2021). Umbrella platform of Tencent eSports industry in China [Article]. Journal of Cultural Economy, 14(1), 9-25. https://doi.org/10.1080/17530350.2020.1788625

Zhao, Y., & Zhu, Y. (2021). Identity transformation, stigma power, and mental wellbeing of Chinese eSports professional players [Article]. International Journal of Cultural Studies, 24(3), 485-503. https://doi.org/10.1177/1367877920975783

Zhou, F., Huang, S., & Liu, C. (2015). Business models in e-sports: Starcraft 2. 15th International Conference on Electronic Business, ICEB 2015,

Zhu, J., Liu, F., Li, Y., Lim, E., Tan, C. W., & Liu, H. (2021). Unraveling the effects of experience-based faultlines in E-sports teams: The moderating influence of team winning momentum. 2020 International Conference on Information Systems - Making Digital Inclusive: Blending the Local and the Global, ICIS 2020,

Zimmer, R. T., Haupt, S., Heidenreich, H., & Schmidt, W. F. J. (2022). Acute Effects of Esports on the Cardiovascular System and Energy Expenditure in Amateur Esports Players. Front Sports Act Living, 4, 824006. https://doi.org/10.3389/fspor.2022.824006
